# Study of Batch and Semibatch Reactive Crystallization of l‑Glutamic Acid with aid of PATs, focusing on Polymorphism and Crystal Habit

**DOI:** 10.1021/acs.cgd.5c01005

**Published:** 2025-10-15

**Authors:** Biyu Zhang, Christos Xiouras, Merve Öner, Georgios D. Stefanidis, Tom Van Gerven

**Affiliations:** a Department of Chemical Engineering, Process Engineering for Sustainable Systems, 26657 KU Leuven, Celestijnenlaan 200F, Leuven 3001, Belgium; b Janssen Research and Development, Janssen Pharmaceutical Companies of Johnson & Johnson, Turnhoutseweg 30, Beerse 2340, Belgium; c School of Chemical Engineering, Department of Process Analysis and Plant Design, 68994 National Technical University of Athens, Iroon Polytecneiou 9, Zografou, Athens 15780, Greece

## Abstract

This work investigates the reactive crystallization of l-glutamic acid under different supersaturation generation strategies. Using PATs such as ATR-FTIR and in situ microscopes enables real-time monitoring of the supersaturation trajectory as well as the nucleation and growth behavior. In the batch process, crystallization starts instantaneously after the acid is added, leading to fines and clusters in the final products. Conversely, in the semibatch process, acid is dosed continuously, and more uniform α-LGA crystals are produced. The stochastic induction points were between the supersaturation range S_ind_ of 3 ∼ 5, and contributed to two distinct crystal habits. There seems to be a critical supersaturation level S_crt_, specific for each certain dosing rate. If S_ind_ is higher than S_crt_, it would enable faster transition of the generated nuclei to large sizes with lower tendency to agglomerate. If S_ind_ is below the value, it can not provide adequate driving force to allow the fast transition and lead to intergrown agglomerates. Moreover, it is found that applying ultrasound during the semibatch processes enhances the crystallization kinetics, making the induction points more reproducible, reducing the agglomeration, and affecting the competition of polymorphic forms. Integrating ultrasound into the hybrid batch/semibatch process could reduce the induction time and produce uniform and nearly pure α-LGA crystals.

## Graphical abstract

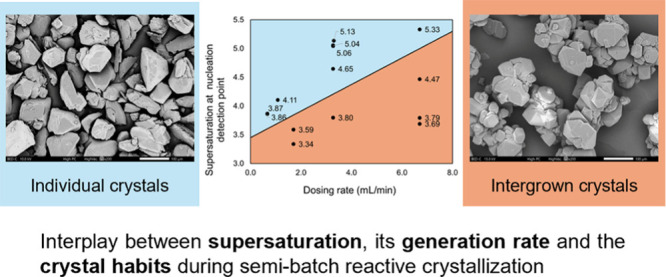

## 1 Introduction

Reactive crystallization processes are widely applied in the chemical and pharmaceutical industries, including the manufacturing of pharmaceuticals and the production of amphoteric compounds, such as amino acids.^1–4^ They are driven by a chemical reaction of freely soluble reagents, which generates a low-soluble product. The supersaturation can be manipulated only by adjusting the reaction rate, making it more difficult to control. The chemical reaction is usually fast, which leads to the generation of extremely high supersaturation levels. Such high supersaturation, implying a high driving force for crystallization, can complicate control of the crystallization process. Therefore, it is critical to control supersaturation for achieving desired crystal properties.^5^ Reactive crystallization processes, often coupling reaction, dilution, and crystallization dynamics, have strong nonlinearities, which make it difficult to employ direct concentration control which is commonly used in other crystallization operating modes, such antisolvent or cooling crystallization.^6^ Teychené et al. reviewed the control of reactive crystallization kinetics by improving the mixing efficiency and using additives.^7^ They pointed out that the ″nonclassical” nucleation behavior could be prominent in most reactive crystallization systems. Alatalo et al. built up a closed-loop control reactive crystallization system for supersaturation and polymorphism control.^8–10^ Yet, much more remains to be carried out to gain a better understanding of reactive crystallization processes.

This study investigates reactive crystallization of l-Glutamic Acid (LGA) as a model compound, chosen for its well-studied polymorphism and corresponding well-defined morphologies. According to the literature, LGA exists in two polymorphic forms,^11^ the metastable α-form characterized by prismatic shapes,^12^ and the stable β-form which can present as needles,^13^ plates or flakes, or flower-like shapes,^14^ depending on the supersaturation level and the crystallization methods. The β-form has a lower solubility in water in the temperature range of 10–55 °C.^15^ Schöll et al. conducted batch reactive crystallization of α-LGA from sodium glutamate with hydrochloric acid to study the nucleation kinetics and showed that ATR-FTIR spectroscopy (attenuated total reflection Fourier Transform technique) is a suitable tool to determine the supersaturation.^16^ They further conducted seeded-reactive crystallization and identified the growth mechanism of α-LGA to be integration-controlled and of birth-and-spread type.^17^ Hatakka et al. conducted seeded reactive crystallization of LGA and found that seeds may suppress the nucleation process.^10^ This study focuses on the effects of supersaturation control on reactive crystallization processes; thus, seeding is not employed. To better understand the effects of supersaturation control, this study explores various operating modes, batch, semibatch, and hybrid batch/semibatch processes - during reactive crystallization of LGA.

Additionally, the study incorporates ultrasound as a process enhancement tool. The application of ultrasound in the field of crystallization was first reported in 1927 by Richards and Loomis.^18^ Over decades, there has been an increasing growth of the number of examples of the application of ultrasound for crystallization, especially for active pharmaceutical ingredients (APIs).^19–22^ Many studies and reviews^23–27^ have summarized the application of ultrasound in crystallization and particle engineering. Specifically, ultrasound has the potential to control the particle size distribution, seen in many cooling crystallization systems, such as paracetamol,^28^ Sodium Acetate,^29^ Adipic Acid,^30^ and Piracetam.^31^ Ultrasound can also help reduce the induction time^32^ and work as an in situ seed generation strategy, seen in cooling crystallization of Adipic acid^30^ and reactive crystallization of an aromatic amine.^33^ However, the effects of ultrasound on crystallization vary from compound to compound, and its impact on reactive crystallization has not been sufficiently studied. In our previous study on ultrasound-assisted reactive crystallization of an aromatic amine,^34^ it was found that ultrasound can potentially increase the crystallization rate, reduce particle size and maintain the crystal form. Experimental examination of the final crystal properties under ultrasound is not enough. The complexity of the reactive crystallization system involving ultrasound requires a comprehensive investigation. A deeper understanding of the ultrasound-assisted reaction and crystallization process and the mechanism behind ultrasonic crystallization will help in making rational use of ultrasound technology in the reactive crystallization process. The inline monitoring with the aid of process analytical technology (PAT) and sensors to obtain qualitative and quantitative data would allow for the analysis of reactive crystallization processes.

Therefore, the primary objective of this study is to examine the reactive crystallization of LGA under different operating modes and to understand how supersaturation generation strategies influence nucleation and crystal growth. A secondary objective is to evaluate the potential role of ultrasound in these processes. To enable real-time process understanding, this study employs PATs, including an ATR-FTIR probe for monitoring solution concentration and an in situ microscopy probe for visualizing crystal formation and growth behavior.

## 2 Materials and Methods

### 2.1 Materials

The main reactant used in this study, monosodium glutamate (MSG, ≥ 99%) was purchased from Thermo Scientific and used as received without further purification. Sulfuric acid (99%, 0.5M) was purchased from VWR, Avantor and used as the other reactant. Deionized water was used to prepare a solution of reactants of defined concentration. The specific amount of MSG was weighed and dissolved in water, filtered using a vacuum filtration to remove any potential insoluble matter, and prepared in the volumetric flask before performing crystallization experiments. The concentration of the MSG solution used in this study was prepared as 1.0 mol/L, and the IR spectra of the initial solution were measured to ensure that the initial concentration was identical.

### 2.2 Crystallization experiments

Reactive crystallization experiments were conducted in a 400 mL glass reactor in an EasyMax 402 thermostat Workstation (Mettler Toledo), equipped with an overhead agitation using a four-pitched blade impeller, a condenser, and a Pt100 temperature sensor, whose measurement was used in feedback control of the reaction temperature. Various process analytical technology (PAT) tools were integrated. BlazeMicro 900 (BlazeMetrics) – , a 14-bit HDR in-process microscope, is used to capture the induction point and monitor the crystal evolution over the reaction and crystallization process. Specifically, turbidity (TU), real-time images, and image-derived chord length and particle counts as a function of time were obtained via a BlazeMicro 900. Attenuated total reflection Fourier transform infrared (ATR-FTIR) spectroscopy is applied to monitor the concentration change in the liquid phase and determine the crystallization end point. The ATR probe allows for the acquisition of liquid-phase IR spectra without influence from solids. All ATR-FTIR measurements in this work were carried out using a TE MCT detector in the ReactIR 700 system (Mettler-Toledo) equipped with a DiComp immersion probe and a diamond as the ATR crystal. Besides, a syringe pump (SP-50 dosing unit, Mettler Toledo) connected to the crystallizer was used to dose acid continuously in semibatch experiments. A pH probe communicating with the workstation was used to control the dosing end of the semibatch process, and the dosing was stopped upon measurement of a pH of 3.1 (the isoelectric point of glutamic acid). Figure 1 gives an illustration of the experimental setup.

**Figure 1:**
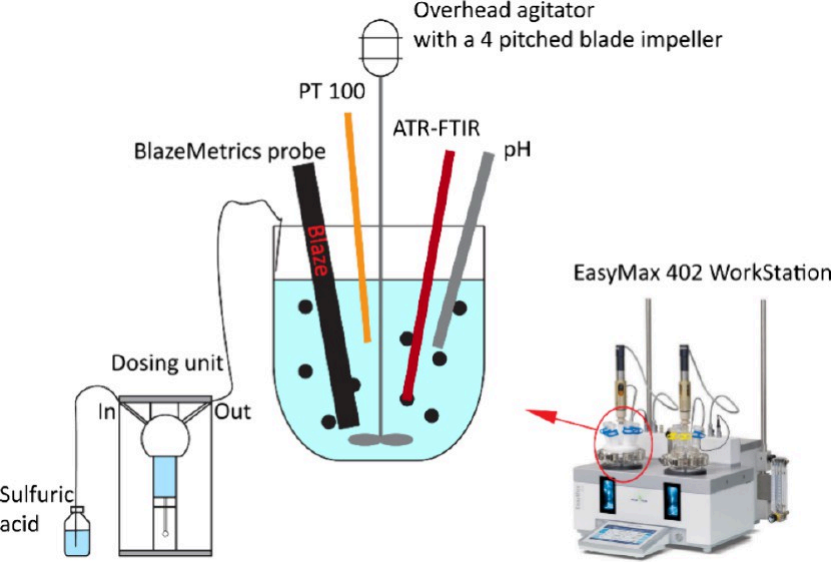
Schematic diagram of the setup used for the reactive crystallization of LGA.

Reactive crystallization of glutamic acid was initialized by adding an equimolar amount of sulfuric acid to the initial MSG solution. The temperature was maintained at 25 °C and the agitation rate was set at 250 rpm for all experiments. As illustrated in Figure 2, different dosing strategies of sulfuric acid, such as batch addition, continuous addition at a constant rate or a two-step rate, and hybrid batch/continuous addition, were applied to generate supersaturation profiles. In a batch process, 200 mL of 1.0 mol/L MSG solution was preheated in a 400 mL reactor. Equimolar sulfuric acid (200 mL, 0.5 mol/L) was added at once via a funnel into the reactor afterward. In a semibatch process, 200 mL of 1.0 mol/L MSG solution was prepared as an initial solution. 200 mL ± 1 mL of 0.5 mol/L sulfuric acid was dosed at a constant rate continuously through the dosing unit. In the two-step semibatch experiments, continuous dosing of acid was carried out until nucleation onset; after holding for 0.5 −1 h, the second continuous dosing with a lower dosing rate was released. In addition, hybrid batch/semibatch experiments were conducted, which consisted first of batch addition of 20% - 40% acid followed by continuous dosing of the remaining acid. The detailed experimental conditions are summarized in Table 1.

**Figure 2:**
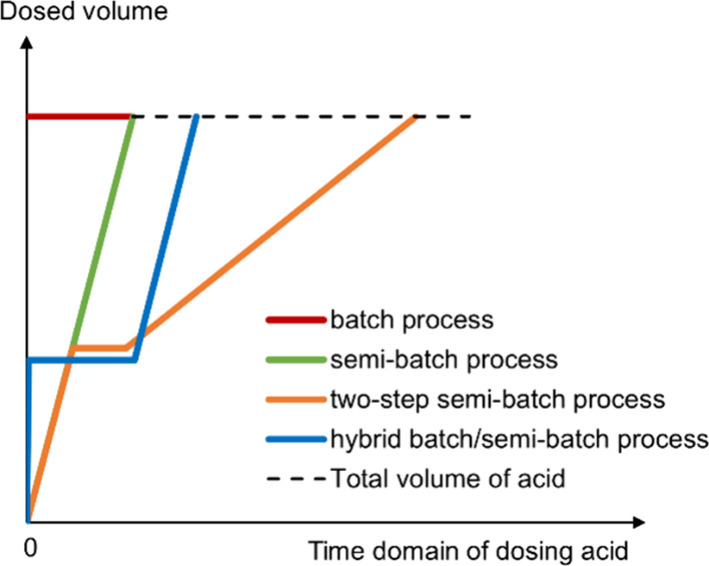
Schematic diagram of the dosing strategies.

**Table 1:** Experimental Conditions and Other Experimental Conditions

experimental conditions	
Dosing strategy[Table-fn t1fn1]	Various dosing rates (mL/min)
Batch	200 mL in one go via a funnel
Semibatch	6.7, 3.3, 1.7, 1.1, 0.7, 0.33
Semibatch with ultrasound[Table-fn t1fn2]	6.7, 3.3, 1.7

other experimental conditions		
Dosing strategy[Table-fn t1fn1]	Dosing rate -1st stage	Dosing rate -2nd stage
Semibatch with two-step rates	6.7 mL/min	1.3 mL/min
	3.3 mL/min	0.7 mL/min
Hybrid batch/semibatch	80 mL in one go	3.3 mL/min
	40 mL in one go	10 mL/min
Ultrasound-assisted hybrid[Table-fn t1fn3]	40 mL in one go, WUS	3.3 mL/min
	40 mL in one go, WUS	1.1 mL/min
	20 mL in one go, WUS	1.1 mL/min

1 Note: The total volume of sulfuric acid used was 200 mL ± 1 mL, corresponding to the controlled end pH of 3.1. The concentration used was 0.5 mol/L, corresponding to 1.0 mol/L H^+^. The same temperature: 25 °C. The same agitation rate 250 rpm.

2 Ultrasound was switched on since acid dosing was started, and stopped at the nucleation onset.

3 Note: Ultrasound was switched on after first batch of acid, and stopped at the nucleation onset.

After the acid dosing finished, the reactor was stirred for 1h to equilibrate. Subsequently, the slurry was filtered and washed using water under vacuum with a Büchner funnel using a filter paper (Whatman 5, diameter 70 mm, pore size 2.5 μm). The collected solids were dried overnight on a Petri dish in a vacuum oven at 40 °C for offline analysis.

### 2.3 Blaze TU-based induction point detection

In this work, the induction time, the time period between the start of acid dosing and the nucleation point, was determined from the turbidity value collected via the BlazeMetrics probe. The detection of the onset of particle formation was considered as the nucleation point in this work. However, the actual nucleation point can be earlier. A fixed threshold method was used to determine the nucleation point in MATLAB. Basically, the Blaze TU data from different experiment runs was normalized and smoothed first. The first derivative of the smoothed TU data was calculated, and the first point at which the TU change exceeded the set threshold was identified as the nucleation point. The determined nucleation point was confirmed by double checking the inline Blaze images. An example is shown in Figure 3.

**Figure 3:**
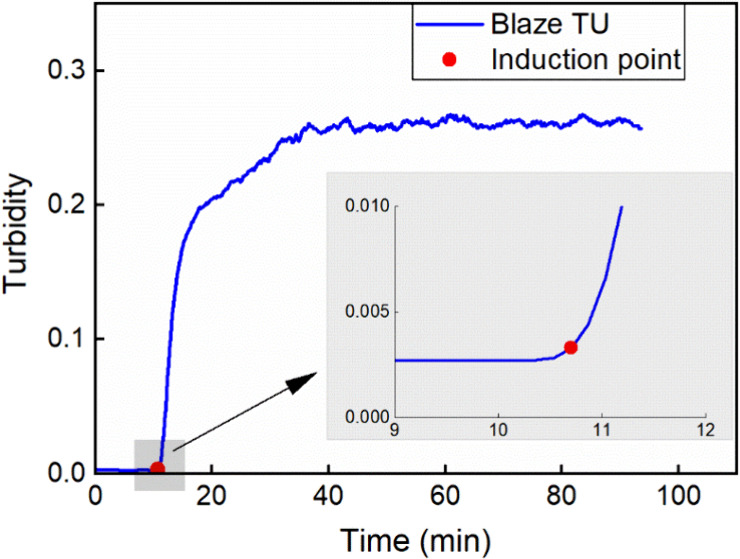
Example of nucleation point detection based on turbidity measurements (raw Blaze TU data shown as a blue line). The red point marks the nucleation point. Detailed data, including the raw turbidity measurements, normalized and smoothed curves, and first derivative results, are provided in Supporting Information S1. (Experimental condition used in this figure: initial MSG 200 mL of 1.0M, dosing sulfuric acid 200 mL of 0.5 M at 6.7 mL/min.).

### 2.4 Calibration of ATR-FTIR spectroscopy

Preliminary experiments, including equilibrium experiments and dynamic crystallization experiments, were conducted to collect data for the calibration of ATR-FTIR spectra to the total concentration of molecule [Glu]. The equilibrium experiment was carried out by the stepwise addition of sulfuric acid to the preheated monosodium glutamate solution. Before and after each addition, the equilibrated IR spectra were collected, and an offline solution sample was taken for concentration determination of total concentration of molecule [Glu] in UPLC (ultraperformance liquid chromatography). Similarly, solution samples from the process during the continuous acid dosing in dynamic reactive crystallization experiments were taken as the known concentration input in the model. In total, nine experiments yielding 75 samples are used to build the IR model in the software iC Quant (Mettler Toledo). The details of the calibration experiments and model information can be found in the Supporting Information S2. The ATR-FTIR probe can measure the spectra of whole wavenumber range of 2500–650 cm^–1^. Considering the noise from solvent (water) is around 1600 cm^–1^ and that carboxylate stretching band is at 1402 cm^–1^, the spectra between wavenumber range of 1500–1000 cm^–1^ is used as input in the calibration model. For iC Quant settings, two-point baseline correction from 1500 cm^–1^ and 1000 cm^–1^, and mean centering methods were applied to process the spectra data and calibrate it against the known concentration based on the partial least-squares regression (PLSR) algorithm. Figure 4 shows a partial set of spectra and the corresponding concentration data used to calibrate the IR model.

**Figure 4:**
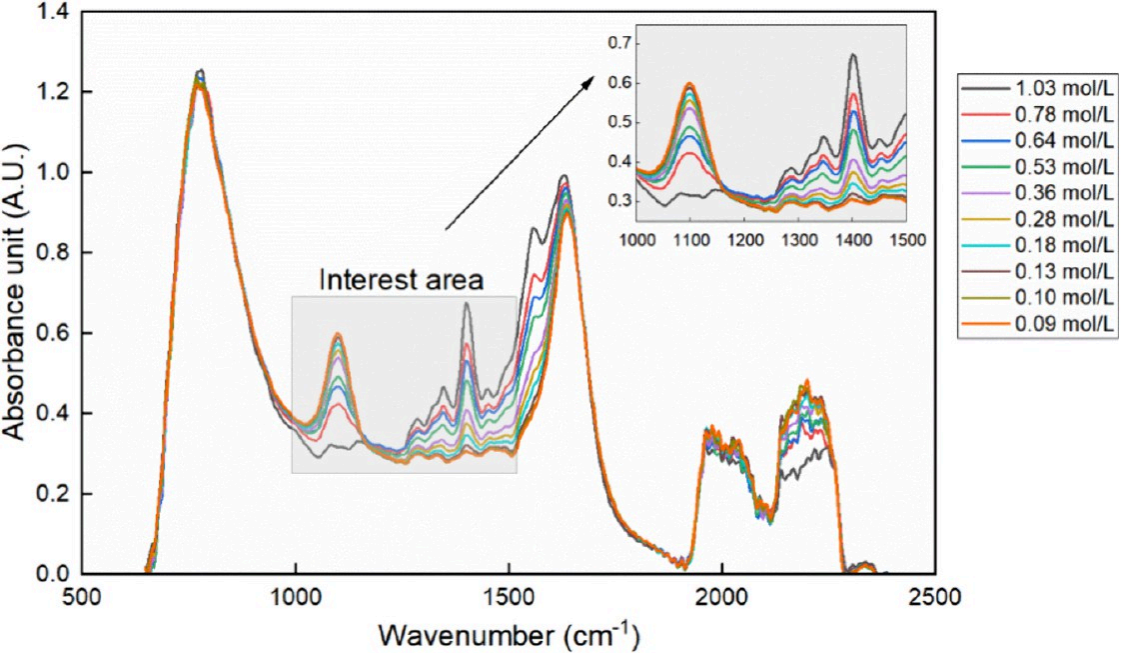
IR spectra with corresponding concentrations used to calibrate the IR model.

It should be noted that both IR and UPLC can only measure the total concentration of [Glu] in the liquid phase consisting of different species [Glu]^+1^, [Glu]^0^, and [Glu]^−1^ in the studied pH range:

$$\left[\mathrm{Glu}\right]={\left[\mathrm{Glu}\right]}^{+1}+{\left[\mathrm{Glu}\right]}^{0}+{\left[\mathrm{Glu}\right]}^{-1}$$

Once the total concentration is determined either by UPLC or IR, the concentration of each species can be calculated based on the dissociation equilibrium at 25 °C^35,36^ and the pH value:

$$H\cdot Glu\left(l\right)↔H^{+}+{Glu}^{-},K_{a}=4.57\times 10^{-5}$$

$${Glu}^{+}\left(l\right)↔H^{+}+H\cdot Glu,K_{a}=6.2\times 10^{-3}$$

$$\mathrm{pH}=-\mathrm{lg}\left[H^{+}\right]$$

The solubility of α-LGA in water at 25 °C is 0.08 mol/L, recalculated from the mole fraction solubility 13.88 × 10^–4^ in pure water at 298 K as reported in literature.^37^ The supersaturation is calculated as

$$S=\frac{c_{{Glu}^{0}}}{c^{*}}$$

where c* and *c*_*Glu* ^0^_ is the solubility of α-LGA at 25 °C and calculated concentration of neutral LGA species *Glu* ^0^, respectively. It should be noted that the supersaturation calculation is enabled by direct association of the driving force with the solute mass balance. A thermodynamically more accurate calculation based on the chemical potential difference can be found in the literature.^38,39^ A representative demonstration of concentration monitoring of each species and supersaturation level change during a semibatch experiment can be found in Figure 5, which shows the process of building up supersaturation before crystallization onset since dosing starts, and the decrease of supersaturation due to the consumption rate over generation rate during the crystallization process.

**Figure 5:**
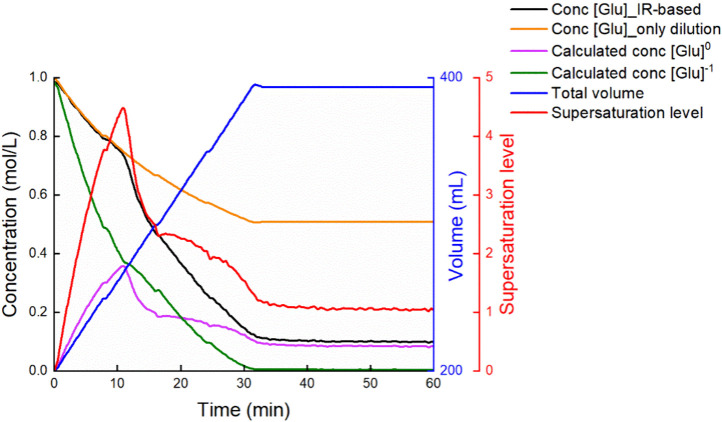
Concentration monitoring of each species and supersaturation level change during a semibatch experiment with dosing rate of 6.7 mL/min. Conc [Glu] refers to the total concentration of molecule [Glu] in liquid phase, which includes the concentration of [Glu]^0^ (purple line), [Glu]^+^ (too low, not shown in this figure) and [Glu]^−1^ (green line).

### 2.5 Offline analysis

Crystal form was examined by X-ray powder diffraction (XRPD) with a diffractometer device Panalytical AERIS (measured range of 2θ: 4° - 50°). The Data Viewer program (Malvern Panalytical) was used to preview the diffraction patterns and export the XRD data for postprocessing. As shown in Figure 6, the characteristic peaks of α-LGA are 15.2°, 17.26°, and 18.28°, while 10.2°, 13.72°, and 17.88° are the characteristic peaks of β-LGA.^40–42^ In this work, “Nearly pure α-form LGA” was used to describe the sample without visible characteristic peaks of β-form LGA, considering the possible measurement limitation of the XPRD device.

**Figure 6:**
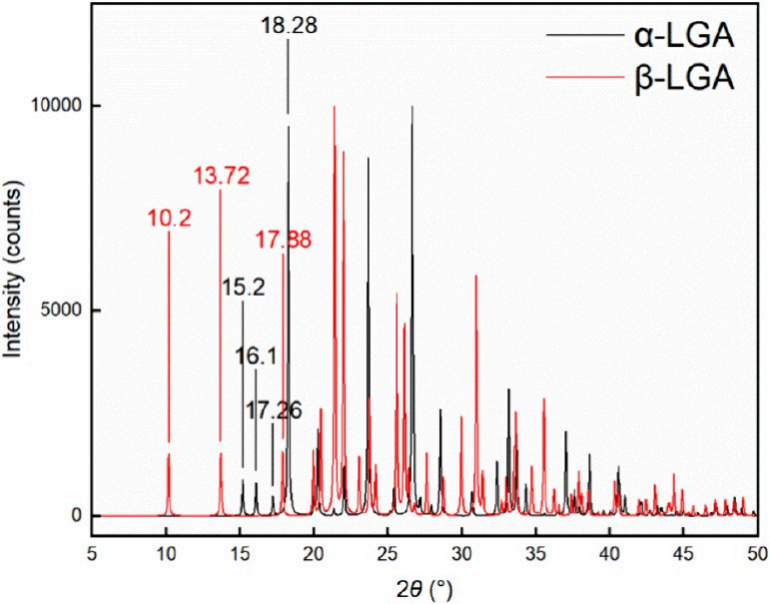
XRD patterns of polymorphs α-Glu and β-Glu.

The morphology of the single crystals and the overall crystal habit of solid samples were determined by scanning electron microscopy (SEM). All samples were gold-coated using a Quorum Q150R S automatic sputter coater and analyzed using a JCM-7000 NeoScope Benchtop SEM device of JEOL. As the polymorphs α and β have distinct morphology as mentioned in Section 1, SEM images could also reveal the potential trace of the existing polymorph, which was not shown in XRD spectra. In this work, the term ‘morphology’ is used to describe the shape of the individual crystal, while the term ‘crystal habit’ means the overall appearance of the final particle. Details about the crystal habits are given in Section 3.2.

The average particle size and size distribution were measured by the laser diffraction technique in a Mastersizer 3000 instrument (Malvern Panalytical) equipped with an automated wet dispersion unit (Hydro MV, Malvern Panalytical). Propanol was chosen as the dispersant due to the low solubility of LGA in it. The stirring speed was set at 1500 rpm. After 10–20 measurements, when the size values D_10_, D_50_, D_90_, and D_99_ reach a stable state, the final result is calculated as the average of the last five measurements for which according to the software (Mastersizer Xplorer software) the standard deviation is less than 0.1%.

### 2.6 Ultrasound condition

A UP200S ultrasonic device (200 W, 24 kHz, Hielscher Ultrasound Technology) with the sonotrode S14L2D (made of titanium, Ø14 mm, approximately length 200 mm, with seal ring FKM) was used in the ultrasound experiments. The minimum 20% amplitude with a corresponding 40 W and 0.5-cycled pulse ultrasound (0.5 s sonication -0.5 s pause) was applied as the ultrasound condition.

## 3 Results

In this work, reactive crystallization experiments were conducted under different dosing profiles of sulfuric acid. Detailed conditions can be found in Figure 2 and Table 1. In the batch experiments, all acid was added in one dosing moment, and the resulting final particle properties were analyzed. In the semibatch experiments, the induction points under different dosing rates were analyzed and reported as induction time together with the corresponding pH, volume of acid, and supersaturation level at these induction points. In the hybrid experiments, the first batch of acid generated the initial supersaturation; then further dosing was halted until nucleation onset, whereafter the remaining acid was continuously dosed to complete the reaction. Final particle properties were analyzed and compared with those from the batch and semibatch experiments. Additionally, by introducing ultrasound to semibatch experiments and hybrid experiments, the impacts of ultrasound were explored.

### 3.1 Batch process

The batch reactive crystallization experiments under the same conditions were conducted twice. In the batch process, nucleation happened immediately after sulfuric acid was added to the reactor. The supersaturation level reached 6.1, and the desupersaturation proceeded afterward, as shown in Figure 7. After holding one h for equilibrium, the slurry was isolated by vacuum filtration. Final dry solids analysis showed that a nearly pure α-form LGA was obtained. The crystal habit is presented in SEM images in Figure 8, from which one can see that all the α-LGA single crystals appeared to be prismatic, although there were some clustered particles. The SEM images exhibited the formation of fines and agglomerates, which was due to a fast reaction and an uncontrolled precipitation process in the batch mode.

**Figure 7:**
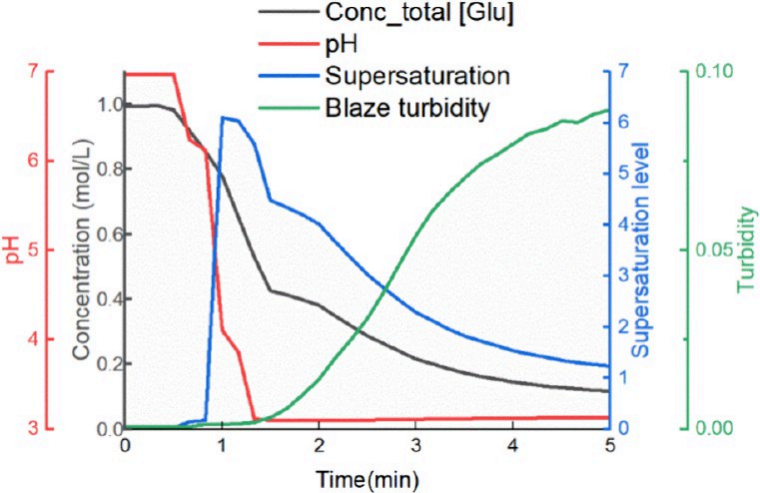
A representative figure showing the concentration, pH, supersaturation, and turbidity monitoring of batch reactive crystallization experiments. The condition used in this figure: dosing 200 mL of 0.5 M sulfuric acid to 200 mL of 1.0 M MSG at once.

**Figure 8:**
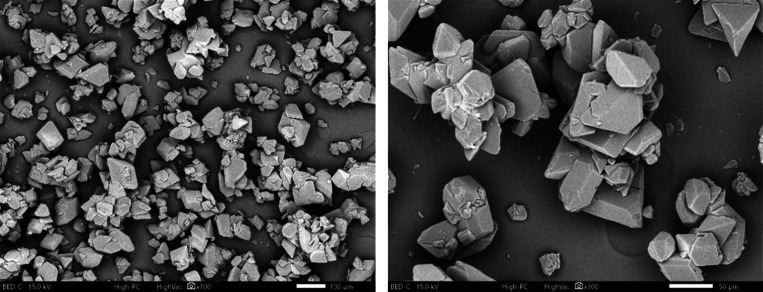
SEM of experimental samples obtained from a batch process at a supersaturation of 6.1.

### 3.2 Semibatch process

In the semibatch experiments, sulfuric acid was dosed continuously at a constant rate, and the supersaturation was built up gradually. Figure 9 presents the parameter monitoring of a semibatch experiment, including total concentration of all LGA species [Glu], pH, supersaturation, and turbidity. One can see that pH decreases over acid dosing, and total concentration decreases because of both dilution as dosing goes on and the precipitation occurring later. The supersaturation first increases due to the reaction generating neutral LGA species and starts decreasing due to the crystallization onset, which corresponds to a turbidity change. Different dosing rates were applied, and the resulting induction points and particle properties were investigated. Figure 10 shows the induction point measurement results under different dosing rates and the corresponding supersaturation level. Each dosing rate was repeated at least twice (except for the case at 1.1 mL/min). It was found that with the increase in the dosing rates, the resulting induction time was shorter, while the consumed volume of acid was between 40 and 80 mL, corresponding to 20% to 40% of the total volume, and the induction pH was 4.1–4.7. The induction points were stochastic in a semibatch process, especially at the higher dosing rates, which may be attributed to local mixing inhomogeneity and transient fluctuations in local supersaturation. At higher dosing rates, supersaturation is generated rapidly, and the added acid may not be fully homogenized before nucleation occurs. Such transient concentration fluctuations are likely to increase the variability of the induction time. Similar effects have been reported by O’Grady et al.,^43^ where faster addition rates of antisolvent led to broader metastable zones and increased induction time variability. Two distinct crystal habits of α-LGA were obtained under the same dosing rates, where α-LGA nucleated at different resulting induction points.

**Figure 9:**
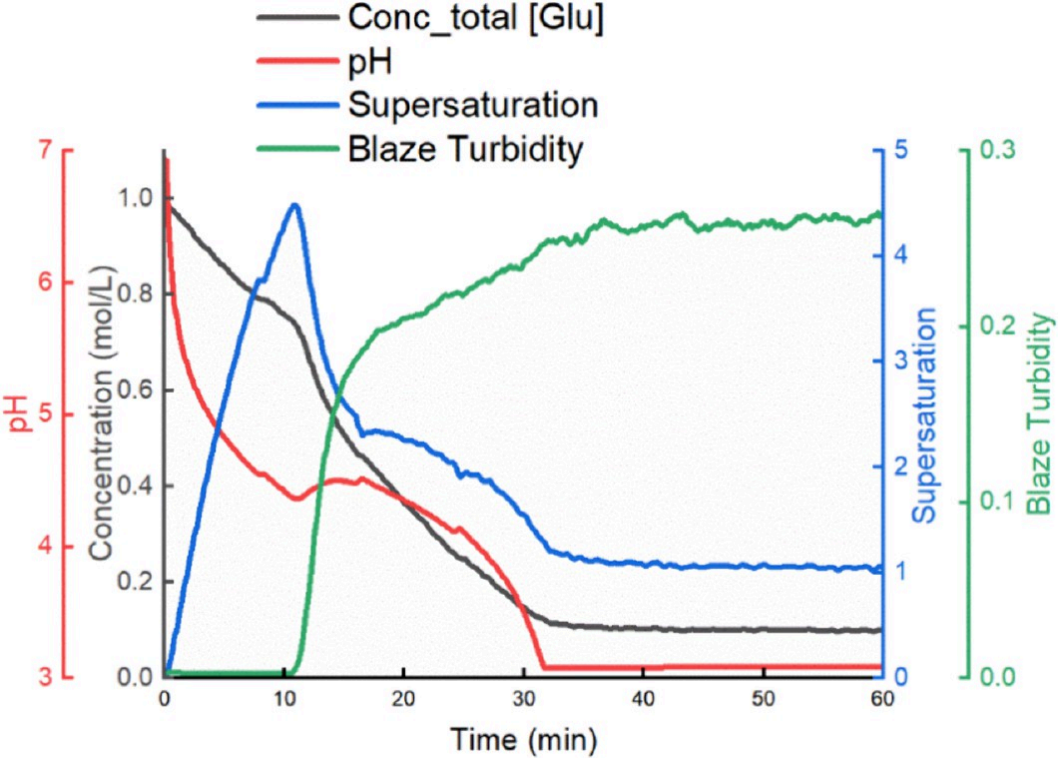
A representative figure showing the total concentration of all LGA species [Glu], pH, supersaturation, and turbidity monitoring of semibatch reactive crystallization experiments (condition used in this figure: initial MSG 200 mL of 1.0M, dosing sulfuric acid 200 mL of 0.5 M at 6.7 mL/min). The detailed concentration changes of different LGA species are listed in Figure 5.

**Figure 10:**
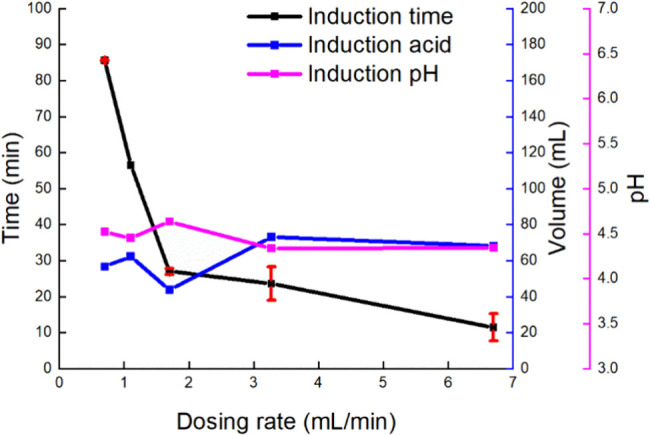
Induction points under various dosing rates in the semibatch experiments. “Induction acid” and “induction pH” labels refer to the volume of consumed acid and the corresponding pH at nucleation onset, respectively.

At the dosing rate of 3.3 mL/min, the induction time was between 15 and 29 min, corresponding to the supersaturation level at nucleation onset (induction supersaturation, written as S_ind_) between 3.8 and 5.1. It was observed from the Blaze inline images that a higher S_ind_ led to very rapid growth of nuclei into larger crystals seen in the beginning and less-agglomerated crystal products in the end, as shown in Figure 11 (a)-(c). A relatively lower S_ind_ of 3.8 led to slower growth of nuclei in the beginning and well-agglomerated crystal products in the end, as shown in Figure 11 (d)-(f). The two distinct crystal habits were examined by SEM and are shown in Figure 11 (g)-(j). One can see that the experiment under high S_ind_ had some big crystals, and the majority were still small, which was consistent with the particle size measurement results. Specifically, the average volume-based sizes were larger and the size distribution was broader in the case of individual crystal habits compared to the case of intergrown agglomerates. XRD analysis confirmed that the products with two distinct crystal habits belonged to the same polymorph of α-LGA.

**Figure 11:**
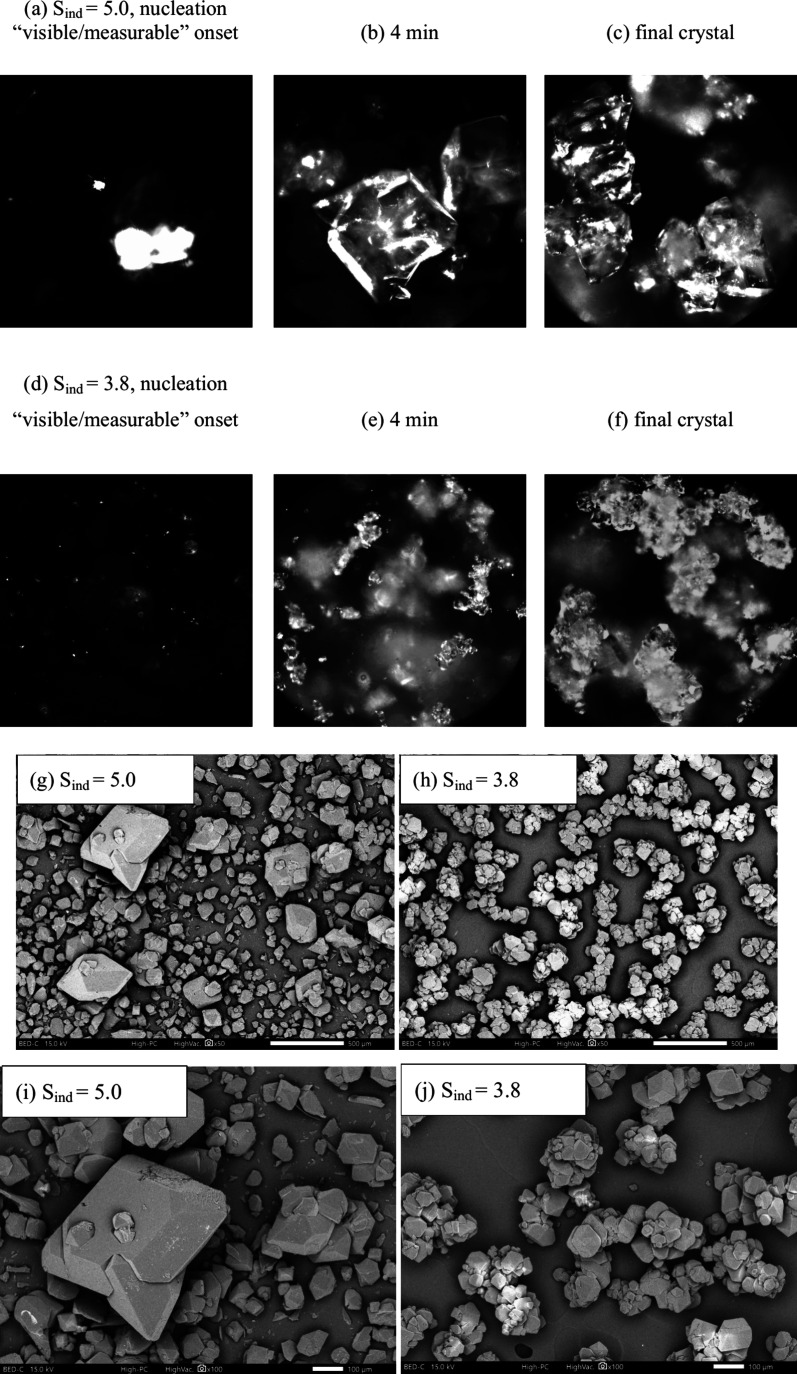
Images (a)-(c) are representative Blaze images captured at the initial nucleation stage, around 4 min after nucleation onset, and the final stage before filtration, respectively, from the experiment with a dosing rate of 3.3 mL/min and S_ind_ of 5.04. Images d-f are from the experiment with the same dosing rate of 3.3 mL/min and relatively lower S_ind_ of 3.80. Images (g)-(h) are the SEM images of the final dry crystal products of each case and images (i)-(j) are the close-up images with higher magnification.

Usually, high supersaturation favors strong nucleation and agglomeration, as seen in the batch reactive crystallization of LGA reported in the literature.^44^ In this study, multiple repetitions of semibatch experiments at different dosing rates were conducted, confirming the pattern of the relationship between the acid dosing rates and the resulting S_ind_, and the crystal habits, shown in Figure 12. Along the vertical direction of Figure 12, at the dosing rates of 6.7 mL/min, higher S_ind_ over 5.0 favored individual crystal habit, and a relatively lower supersaturation level below 4.5 led to an intergrown agglomerates habit. Similar findings were seen at the dosing rate of 3.3 mL/min. At the lower dosing rates of 1.1 mL/min and 0.7 mL/min, a supersaturation level over 3.9 led to rapid growth of nuclei and finally less-agglomerated habit. Along the horizontal direction of Figure 12, one may notice that different crystal habits were achieved at the same S_ind_ level generated by different dosing rates. For instance, at the same S_ind_ of 4.6, the high dosing rate of 6.7 mL/min led to intergrown crystals, while relatively low dosing rates below 5.0 mL/min led to individual crystals. Similarly at S_ind_ of 3.8, the high dosing rate of above 2.0 mL/min led to intergrown crystals, while the relatively low dosing rate of 0.7 mL/min led to individual crystals. Therefore, as the dosing rates increased, the supersaturation level S_ind_ favoring rapid crystal growth increased as well. Higher dosing rates could more likely lead to intergrown crystals, which implied that the different crystal habits were not simply driven by supersaturation levels in the traditional way. Both supersaturation levels and supersaturation generation rates influence the competition between nucleation and growth. A consistent and reproducible pattern was found that high supersaturation favored the formation of larger crystals in the beginning and consequently less-agglomerated crystals. One hypothesis is that at a particular dosing rate, a critical supersaturation level, S_crt_, exists, shown as the apparent boundary of two crystal habits shown in Figure 12. In the case that S_ind_ is above S_crt_, it favors the rapid growth of nuclei, leading to large individual crystals in the nucleation stage. The relatively low supersaturation S_ind_ (below S_crt_) means insufficient driving force for rapid growth in the beginning, followed by collision of small crystals and consequently intergrown crystals on the defects caused by collision. It should be noted that S_ind_ is not a predefined experimental condition but a stochastic nucleation outcome, which means that it cannot be directly controlled. Accordingly, S_crt_ is regarded as a descriptive concept to help understand the relationship between dosing rates and crystal habits rather than a physical parameter for crystal habit prediction. The desupersaturation profiles under 6.7 and 3.3 mL/min are shown in Figure 13. In the cases ending with individual crystals, the relative duration during which supersaturation stayed within 90% - 100% of its peak value was longer. In the cases with intergrown agglomerates, a more rapid onset of the decline in supersaturation was observed, which suggested that intergrown growth of many small crystals consumed more supersaturation than fast growth of few large individual crystals. This could be due to that intergrown growth of small crystals at nucleation onset requiring more crystal surfaces. On the other hand, a high supersaturation level above the critical supersaturation level maintained a high level for a while (longer supersaturation plateau in Figure 13), which maybe due to the fast growth of single crystals with less surface area required. Afterward, it dropped rapidly due to secondary nucleation and crystal growth, finally leading to the crystal habit of both large and small individual crystals with very limited agglomeration. Please note that the absolute decrease rates of supersaturation level were not comparable due to the fact that a higher initial supersaturation level was expected to induce a higher decrease rate of supersaturation. Therefore, the relative duration during which supersaturation stayed within the 90%- 100% plateau was considered more representative.

**Figure 12:**
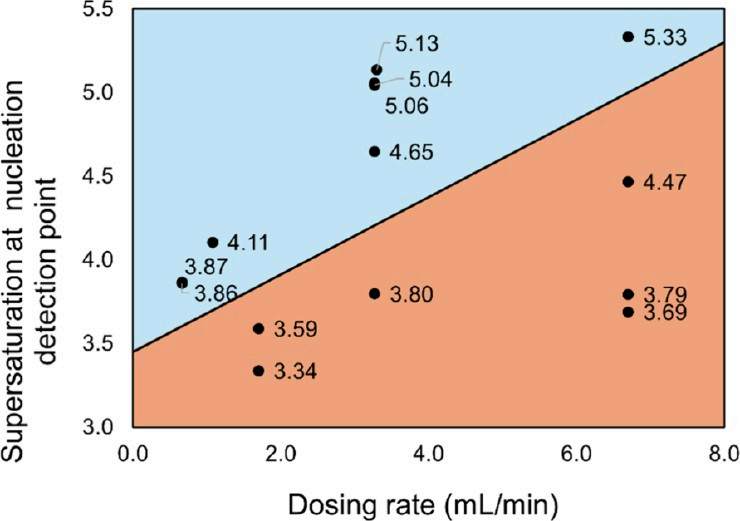
Schematic diagram indicating the supersaturation level at nucleation onset under different dosing rates (shown as black round points with the data labeling the S_ind_ value). Two distinct regions represent the two crystal habits – the case of individual crystals in the light blue region and the case of intergrown agglomerates in the light orange region. The boundary line between two regions represents the critical supersaturation S_crt_. S_crt_ is not a physically defined parameter but a schematic boundary drawn to illustrate the differences between the two observed regions.

**Figure 13:**
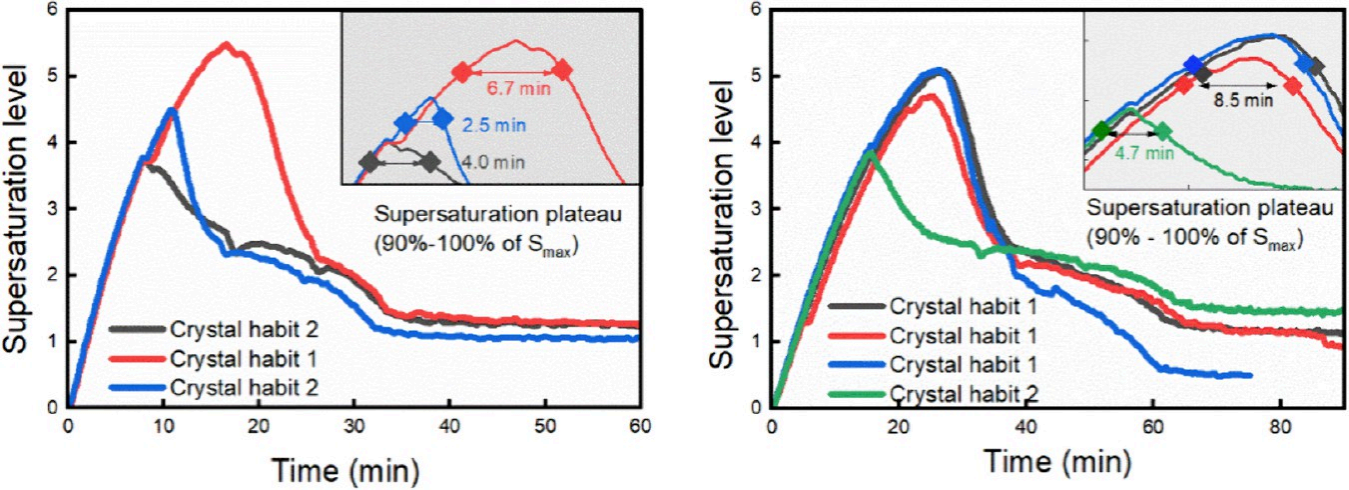
Desupersaturation profiles under dosing rates of 6.7 and 3.3 mL/min (right). The dots in the shaded region show the supersaturation plateau within 90%- 100% of its peak value. ‘Crystal habit 1’ and ‘crystal habit 2’ refer to the individual crystal habit and intergrown agglomerated habit, respectively. Most experiments ended up with a supersaturation level of 1 more or less. The experiment with a final supersaturation level <1 (blue curve in the right figure) was due to the measurement error of concentration based on IR.

During the semibatch process, the supersaturation level at induction points S_ind_ was not a predefined variable but the resulting parameter, which remained stochastic, especially at higher dosing rates. The possibility of changing crystal habits was investigated by decreasing the dosing rates after the nucleation onset and the consumption of initially generated supersaturation. Two experiments were conducted at the following: (1) initial dosing rate of 6.7 mL/min until nucleation at S_ind_ = 3.79 and continued dosing rate of 1.3 mL/min after consuming the initial supersaturation; 2) initial dosing rate of 3.3 mL/min until nucleation at S_ind_ = 5.13 and continued dosing rate of 0.7 mL/min after consuming the initial supersaturation. Figure 14 shows both the process images from inline BlazeMicro and the final SEM images of the two cases. Initially, the first case fell into the ‘intergrown agglomerates’ orange region, and the second case fell into the ‘large individual crystals’ blue region, included in Figure 12. Remaining acid was dosed after both the IR peak intensity and Blaze turbidity reached a steady state condition, which confirmed the initially generated supersaturation was consumed. It should be noted that the supersaturation at the steady state was higher than 1, which may be due to the solubility under partially neutralized conditions (i.e., in the presence of unreacted MSG and produced sodium sulfate) being higher than the reference solubility value in pure water used for the calculation. Previous studies have shown that dicarboxylic amino acids such as glutamic acid exhibit salting-in behavior in electrolyte solutions, resulting in enhanced solubility.^45^ Additionally, amino acid solubility has been reported to vary with solution composition due to ion-specific and self-titration effects.^46^ Although precise solubility values are difficult to determine in reactive crystallization systems due to evolving composition, the pure water solubility value is used as a consistent reference throughout this work.

**Figure 14:**
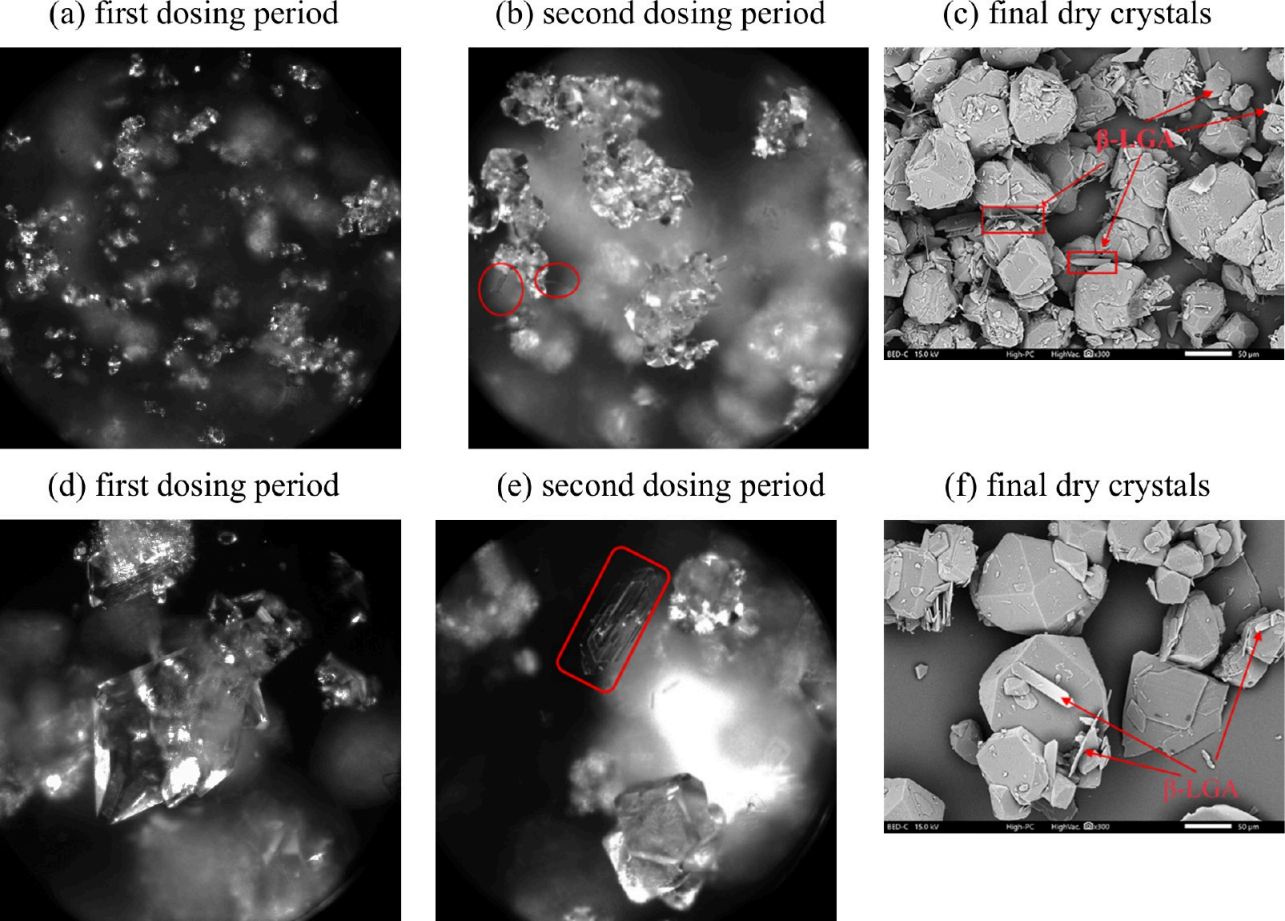
(a)-(b) (d)-(e) BlazeMicro inline images captured during the initial crystallization stage and the second slower dosing period, and (c, f) SEM image of the final crystals under the condition of (1) initial dosing rate of 6.7 mL/min until nucleation at Sind = 3.79 and continued dosing rate of 1.3 mL/min after consuming the initial supersaturation (c) and (2) initial dosing rate of 3.3 mL/min until nucleation at Sind = 5.13 and continued dosing rate of 0.7 mL/min after consuming the initial supersaturation (f). The red marks in the images indicate the presence of β-LGA.

It was observed that the initially formed crystal habits did not change over the second dosing period, while the polymorph profile was affected: the β-form appeared during the second dosing period. One could see from Figure 14 that needle-like and plate-like β-LGA appeared on the surface of α-LGA. Moreover, the desupersaturation profiles were analyzed and compared with the previous semibatch crystallization experiments, as shown in Figure 15. In the experiments with constant dosing rates, the supersaturation levels rapidly decreased to 2.5 and then gradually decreased to the final level of 1.0 gradually. In the experiments with two-step dosing rates, after dosing was paused, the supersaturation level rapidly decreased to around 1.5. During the slower second dosing period, the supersaturation increased a bit to 2.0 and slowly decreased to a final level of 3.1. The slower dosing and consequently longer duration when the supersaturation level was maintained at a relatively low level of 2.0 ∼ 1.0 might have caused the formation of β-LGA. Confirmed by the Blaze inline images, the needle-like β-LGA did not form in the early crystallization stage but in the later long-time dosing period. Since β-form is the thermodynamically stable form, the appearance of β-LGA was likely to come from the solution-mediated conversion – during long-time holding, α-LGA partly dissolved, and then β-LGA nucleated on the surface of α-LGA. Similarly, Roelands et al.^13^ reported that during the LGA precipitation process, the needle-like morphology of β-LGA usually comes from the conversion of α-LGA, not from precipitation. This also explained the results of the semibatch experiments, where trace needle-like β-LGA was seen only during two long runs (300 min under 0.7 mL/min and 600 min under 0.3 mL/min, respectively). The two-step semibatch experimental results suggested that the crystal habits, either with dominance of individual crystals or of intergrown agglomerates, were decided during the initial crystallization stage under prementioned specific conditions of these experiments. Slower dosing after the first supersaturation was consumed did not prevent agglomeration seen in nucleation at S_ind_ = 3.79 experiment, therefore did not have distinct effects on the final crystal habits.

**Figure 15:**
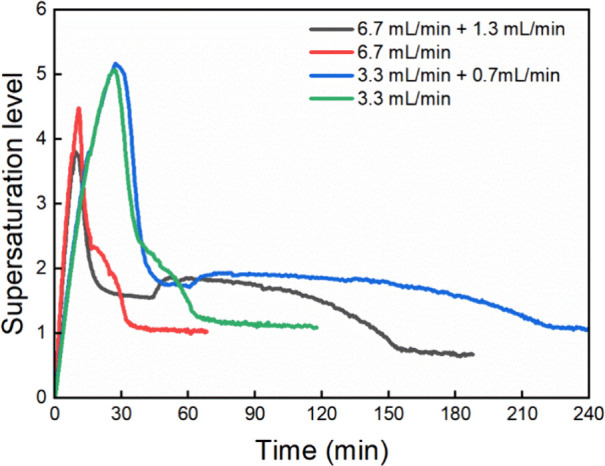
Desupersaturation profile comparison of semibatch experiments with constant dosing rates and with two-step dosing rates.

### 3.3 Hybrid batch/semibatch process

Based on the observations from previous experiments, the dosing rates and induction supersaturation levels significantly affected the crystallization behavior and final crystal habits. In this section, a hybrid batch/semibatch process was conducted, using batch addition of a portion of the sulfuric acid (the same concentration as previous experiments) to generate predefined supersaturation S_ind_ for nucleation, followed by continuous addition of remaining acid to the end. Three trials were conducted, with the first batch addition of (a) 40% acid (80 mL of total 200 mL), (b) 20% acid (40 mL of total 200 mL), and (c) 10% acid (20 mL of total 200 mL), respectively. In Trial (a), the supersaturation level reached 4.8, and nucleation started after 5 min, resulting in small crystals and intergrown agglomerates. In Trial (b), the supersaturation level reached 2.7, and nucleation started after 30 min, resulting in the same agglomeration as Trial (a), shown in Figure 16. Nucleation did not happen even after 24 h in Trial (c); thus, no solid products could be collected for analysis. Compared to the batch case of Section 3.1, where 200 mL acid was dosed at once (instantaneous nucleation with a supersaturation level of 6.1), 40% batch acid generated a supersaturation level of 4.8, leading to induction time of 5.7 min. After holding until Blaze TU reached a stable result, the supersaturation level decreased to 2.0, and the remaining acid was continuously dosed to the system at a dosing rate of 3.3 mL/min for Trial (a) and 10 mL/min for Trial (b), increasing the supersaturation level, as shown in Figure 17. In the end, the two hybrid trials produced mainly intergrown agglomerates. However, compared to the intergrown crystals obtained in the semibatch experiments (Figure 11), the hybrid process led to more individual small crystals, especially combined with fast dosing at 10 mL/min, confirmed by the PSD measurements shown in Figure 17 (right). This indicates that the increased supersaturation level caused by dosing the remaining acid may lead to secondary nucleation and the formation of individual crystals.

**Figure 16:**
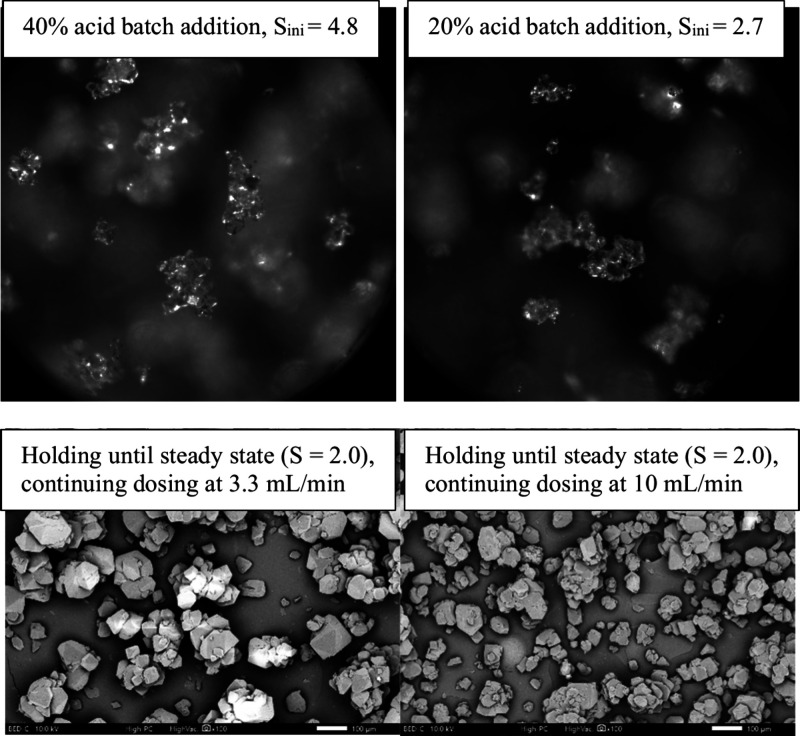
Process images after batch addition (top, captured by Blaze) and SEM images of final dry crystals (bottom) in hybrid batch/semibatch crystallization experiments.

**Figure 17:**
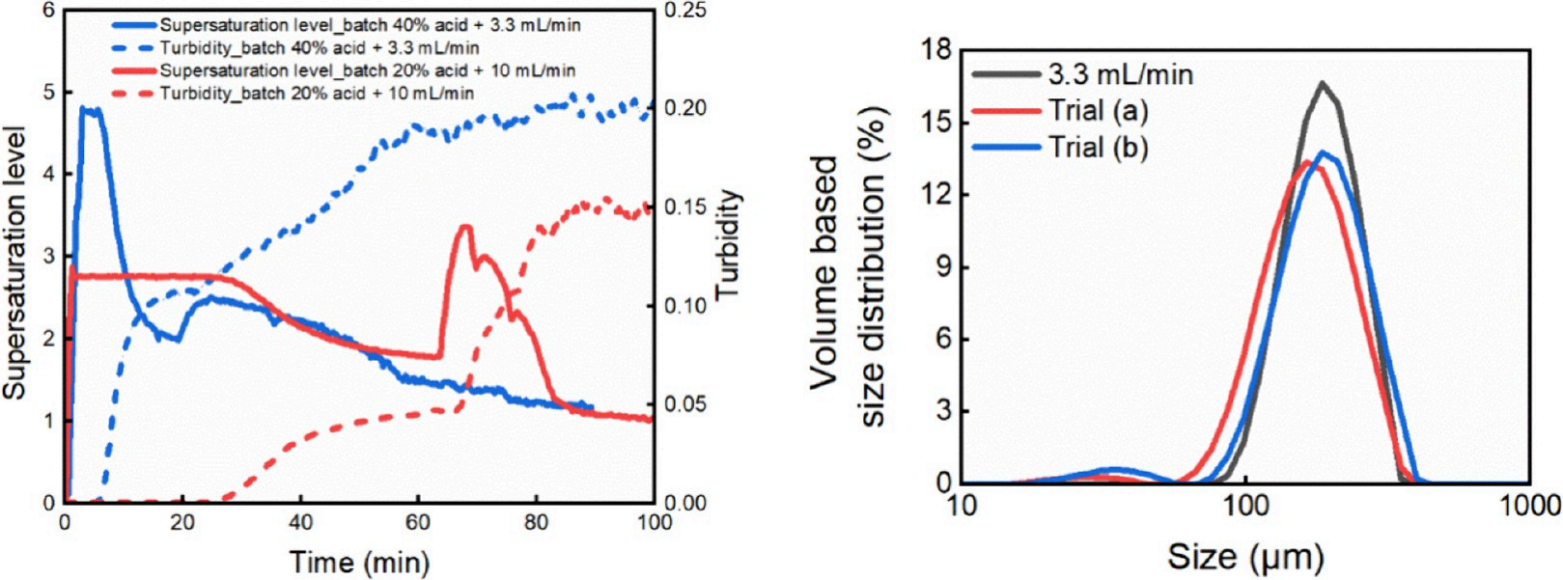
Left desupersaturation profile of hybrid batch/semibatch experiments: trial (a) batch 40% acid +3.3 mL/min, and trial (b) batch 20% acid +10 mL/min. Trial (c) batch 10% acid was not included in the figure due to no crystallization happening after 24 h. Right: PSD comparison of intergrown particles obtained from semibatch experiment under 3.3 mL/min, and hybrid experiments of Trial (a) and Trial (b).

In the reactive crystallization experiments, the supersaturation was generated along the acid dosing duration and consumed after crystallization started. In the batch process ([Sec sec3.1]), the crystallization instantly started after addition of all acid at once, and the supersaturation decreased rapidly in around 5 min, leading to uncontrolled precipitation of crystals. In the semibatch process with a constant dosing rate (Section 3.2), the supersaturation was built up gradually and decreased continuously, leading to better-controlled crystallization behavior and well-structured final crystals. In the two-step semibatch experiments, the dosing rate was changed to a lower level while the final crystal habits were maintained over the second slow dosing period. In the hybrid experiments, the initial supersaturation that was generated by the batch addition of a portion of the acid, decreased since nucleation onset, then increased again due to the second fast addition of acid, which led to the large intergrown agglomerates mixing with small individual crystals. Comparing the supersaturation profiles shown in Figure 17 (left) with the ones in Figure 15 and combining the crystal habit images in Figure 16 and Figure 14, one can conclude that changing dosing rates could affect the crystal habit only if it increased the supersaturation level to a great extent.

### 3.4 The role of ultrasound

In the previous semibatch experiments, it was found that the final crystal habits were dependent on the supersaturation levels at nucleation onset. However, the induction points remained stochastic, especially at high dosing rates of 6.7 and 3.3 mL/min. Ultrasound, as an intensification tool, was reported to have the potential to control nucleation in the field of crystallization. In this section, ultrasound was introduced to semibatch reactive crystallization of LGA. The effects of ultrasound on the induction point and the final crystal habits were investigated by applying ultrasound between the beginning of acid addition and nucleation onset. Additionally, the deagglomeration effects of ultrasound on the intergrown LGA crystals were evaluated by applying ultrasound to the saturated slurry.

#### 3.4.1 Effects on induction time

Before dosing acid to the initial MSG solution, the effects of ultrasound on the temperature, pH, IR spectra, and Blaze information including turbidity value and images were examined. By the temperature control of the jacket, the temperature was well maintained at 25 (+0.5) °C. There were no distinct effects on the pH and IR spectra. In the clear initial solution, the burst of ultrasound generated cavitation bubbles, which slightly increased the turbidity. The turbidity rapidly reached a stable state rapidly. Therefore, the influences on those in-line probes were considered to be negligible. The results are shown in Supporting Information S3.

First, ultrasound-assisted semibatch reactive crystallization experiments were conducted, in which ultrasound was switched on manually at the same time as the dosing of the acid. Right after the nucleation was initialized, ultrasound was stopped, while acid dosing was continued. The ultrasound-assisted induction time results are given in Figure 18. Overall, the semibatch experiments with ultrasound exhibited shorter induction time and appeared more reproducible, which can be seen from generally lower standard deviation of repeated experiments (despite minor differences observed at the lowest dosing rate (0.8 vs 1.3 min)). At the dosing rate of 3.3 mL/min, the application of ultrasound reduced the average induction time by 37.6%, from 23.6 to 14.7 min. The average induction time at the dosing rate of 6.7 mL/min decreased from 11.5 to 9.4 min, corresponding to a 18.1% reduction. The possible explanation is that at higher dosing rates, supersaturation is generated so rapidly that the induction time is already near-minimal, leaving little room for ultrasound to accelerate nucleation further. In semibatch dosing processes, supersaturation generation rate can be considered as an “effective supersaturation level over time”. In this sense, the result aligns with the sonocrystallization reports suggesting that ultrasound most effectively reduces induction time at lower or medium supersaturation.^47,48^ By contrast, at the lowest dosing rate of 1.7 mL/min, the acid feed volume required to induce nucleation (∼44 mL of consumed acid) appears to represent a minimum threshold under certain conditions (initial MSG 200 mL of 1.0 M, reacting with continuously dosed sulfuric acid of 0.5 M, at 25 °C and 250 rpm). Therefore, ultrasound provided only a small additional benefit (∼1.9 mL of reduction in the consumed acid, 7% reduction in induction time). Consistently, the standard deviation of induction times was also lowest at this low dosing rate for both sonicated and conventional experiments, supporting the explanation that nucleation here is governed by a reactant-required threshold rather than ultrasound effects. Furthermore, the reduction rate may also be limited by the current ultrasound settings that may not significantly reduce induction time at certain dosing rates; higher ultrasonic energy input might be able to reduce induction time further.^47,49,50^

**Figure 18:**
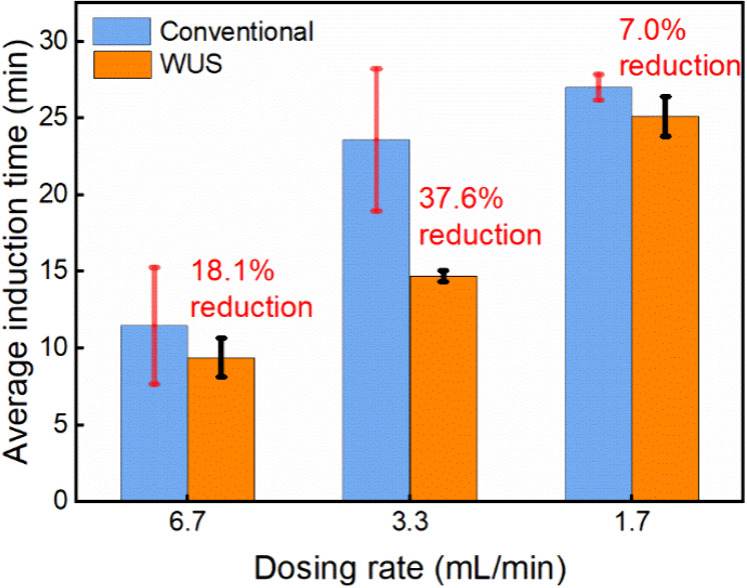
Induction time results of ultrasound-assisted semibatch experiments are shown with the orange bar. The blue bar data show the conventional results, as presented in section 3.2. The error bar shows the standard deviation of at least three repetitions.

**Figure 19:**
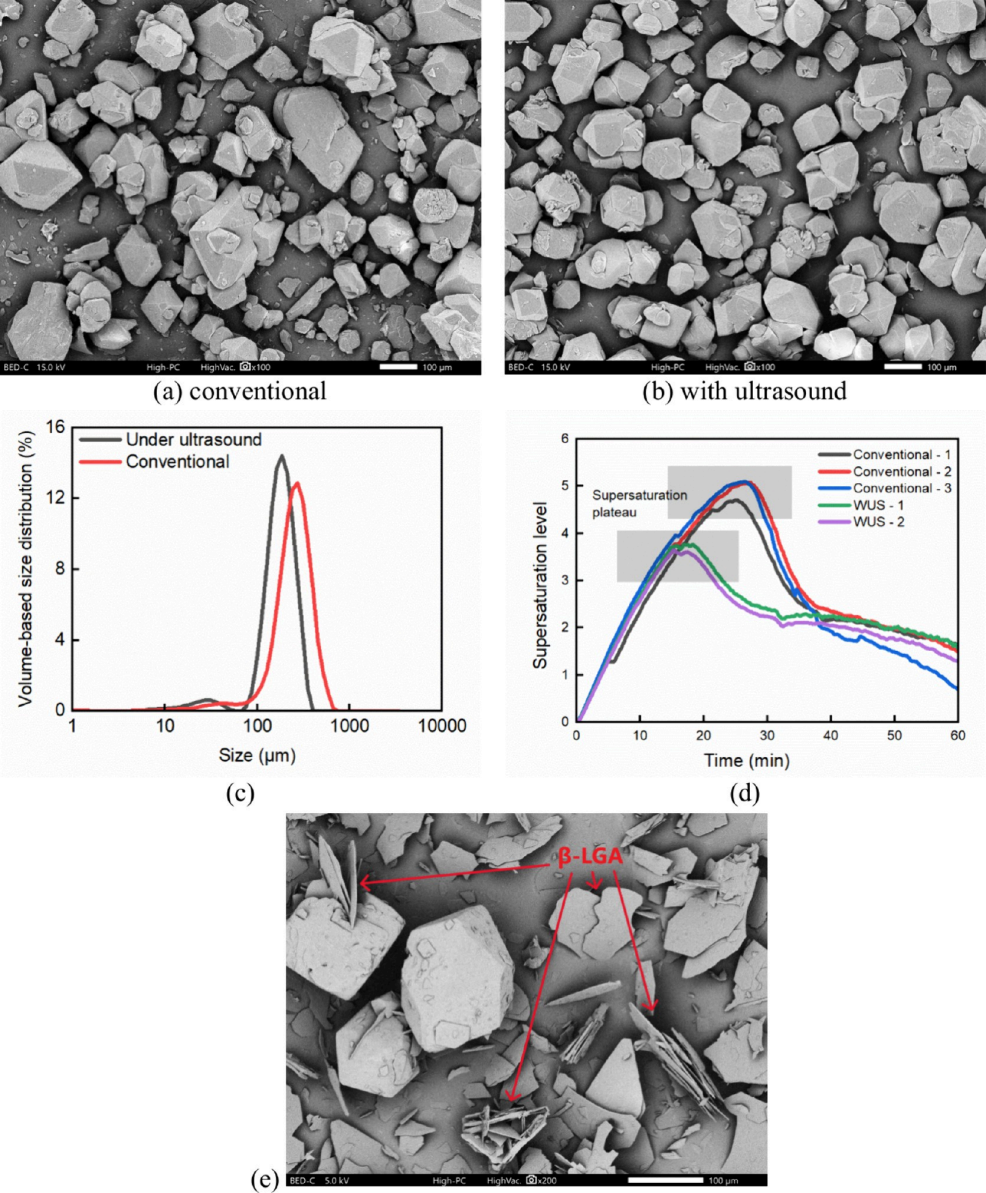
(a) – (b) are representative SEM images of individual α-LGA crystals obtained from conventional and ultrasound-assisted semibatch experiments at the same dosing rate of 3.3 mL/min, respectively. (c) shows the PSD of the experiments producing individual α-LGA crystals under conventional conditions (average of three cases) and sonicated conditions (average of two cases), and their desupersaturation profiles are shown in (d). (e) shows the example of a mixture of α and β produced from ultrasound-assisted semibatch experiments at 3.3 mL/min. The red marks indicate the presence of β-LGA.

**Figure 20:**
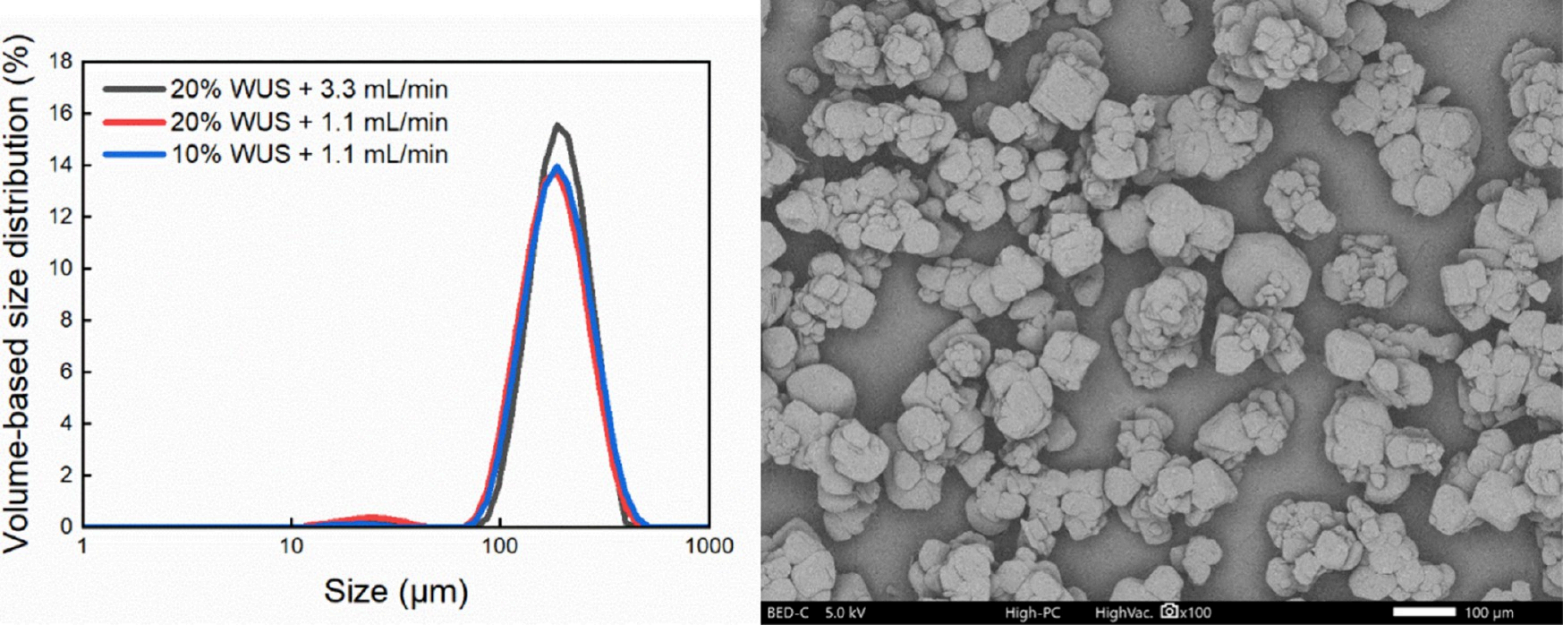
Particle size distribution of the crystals obtained from ultrasound-assisted hybrid experiments (left figure). The legend “20% WUS + 3.3 mL/min” means ultrasound was applied after the first 20% batch acid was added to induce nucleation, then continued dosing at 3.3 mL/min. A representative SEM image from the experiment “20% WUS + 3.3 mL/min” (right figure).

**Figure 21:**
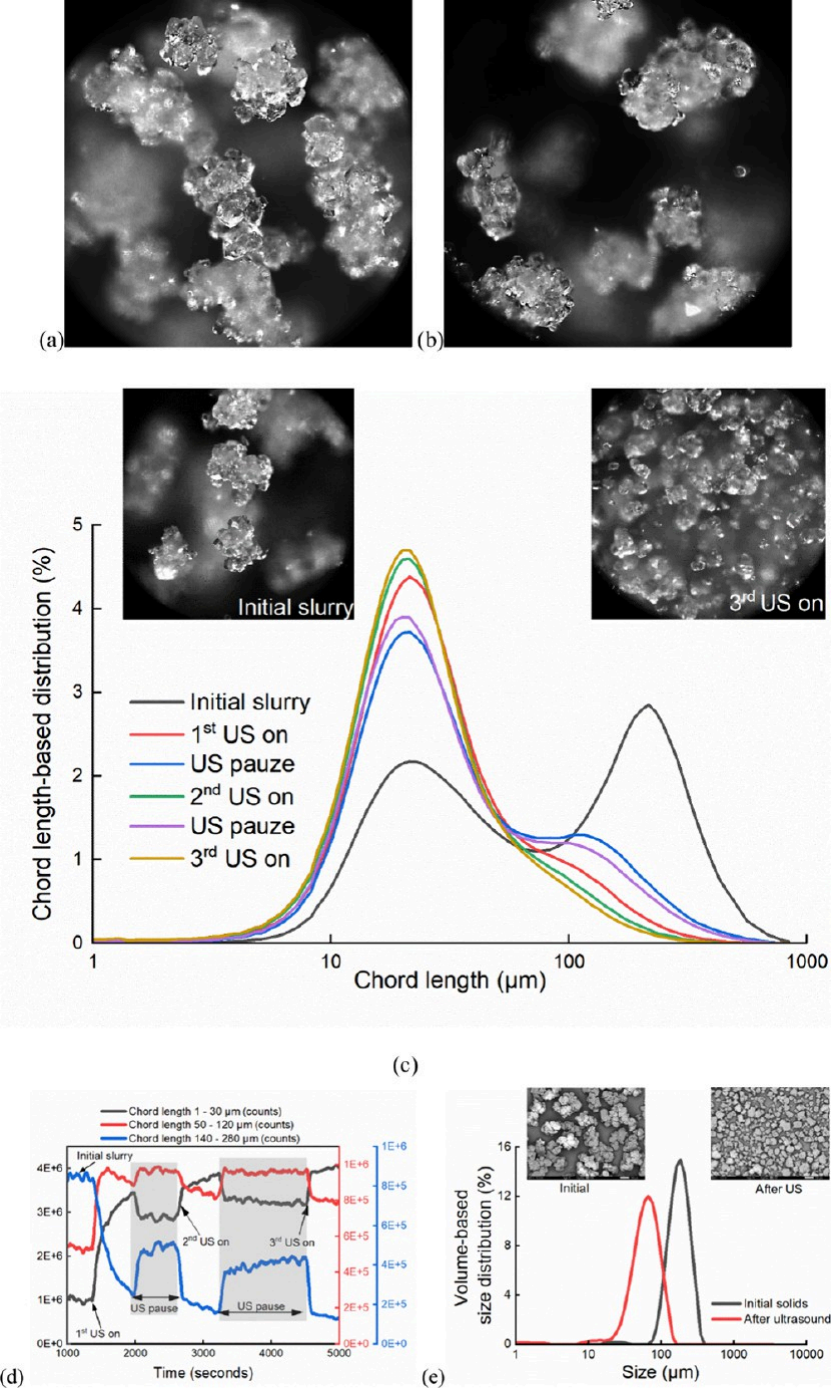
(a) - (b) BlazeMicro images of the slurry from which the collected crystals are produced, and the slurry prepared for the deagglomeration tests using the collected crystals, respectively. (c) Representative process images and CLD trends demonstrating the deagglomeration process by ultrasound. (d) Particle count trends during three cycles of ultrasound on–off. (e) Final PSD results, with corresponding SEM images.

Induction time was reduced, indicating that nucleation happened at a lower supersaturation level in the presence of ultrasound during the semibatch process. The final dry crystals were analyzed to find out the effects of the supersaturation level at ultrasound-induced induction points on the polymorphism and crystal habits, as summarized in Table 2. At a dosing rate of 6.7 mL/min, nearly pure α-LGA crystals were obtained in three experiments, with one case of individual crystals and two cases of intergrown agglomerates. At the dosing rate of 3.3 mL/min, individual α-LGA crystals were obtained in two experiments, while a mixture of α- and β-forms composed predominantly of α-LGA was obtained in two other experiments. The XRD spectra of mixture LGA can be found in Figure 22 as mixture sample 2. The individual α-LGA crystals obtained in sonicated experiments were more uniform than the conventional cases, as shown in Figure 19 (a)-(c). At the dosing rate of 1.7 mL/min, both α and β forms crystallized in one case, while the other two cases ended up with nearly pure α-LGA. It seemed that ultrasound is able to induce individual crystal habits at relatively low supersaturation, which was not observed in conventional experiments. Recalling the fact that all the conventional semibatch experiments produced nearly pure α-LGA under dosing rates from 6.7 to 1.7 mL/min, there seems to be a kinetic advantage of the α-form over the β-form. In the sonication experiments, ultrasound was applied from the start of acid dosing until the onset of nucleation. Under these conditions, the primary effect of ultrasound is expected to be on nucleation rather than the overall crystallization kinetics. The cavitation bubbles can generate localized extreme bubble pressure and temperature,^27^ which promote molecular assembly into critical nuclei, and the high pressure gradients associated with bubble collapse may help the formation of crystal clusters.^51^ Both are suitable for starting the crystallization. On the other hand, ultrasound may also enhance heterogeneous nucleation,^19,48^ with cavitation bubbles acting as nucleation sites, which reduce the effective surface energy and lower the activation energy barrier for nucleation. These combined effects could explain the shorter induction time in sonicated cases and slightly higher occurrence of β-LGA nuclei in some experiments. Blaze inline imaging confirmed that β-LGA appeared during the initial nucleation stage rather than via transformation. Once nucleation began and ultrasound was turned off, the kinetically favored metastable α-form dominated the growth process, leading to a final mixture product with mostly α-form (XRD spectra in Figure 22). Therefore, ultrasound-induced nucleation appears to primarily influence the probability of a nucleated polymorphic outcome rather than altering the growth kinetics of either form.

**Table 2:** Polymorphic form and crystal habit results of ultrasound-assisted semi-batch experiments

Dosing rate (mL/min)	Nucleation onsets information				Polymorphic form based on XRD	Final crystal habit based on SEM
	Induction time (min)	Volume of acid added (mL)	pH	S_ind_		
6.7	10.7	63.4	4.4	4.4	Nearly pure α	Individual crystals
	9.8	57.9	4.5	4.1		Intergrown agglomerates
	7.7	50.0	4.6	3.6		
3.3	14.7	48.5	4.6	3.7	Nearly pure α	Individual uniform crystals
	14.4	47.3	4.6	3.5		
	14.4	47.9	4.6	3.4	Mixture of α and β	Mixture of large α and cluster β
	15.3	50.0	4.6	3.7		
1.7	25.7	42.7	4.6	3.7	Mixture of α and β	Mixture of large α and cluster β
	26.3	43.5	4.6	3.7	Nearly pure α	Intergrown agglomerates
	23.3	39.5	4.7	3.1		

**Figure 22:**
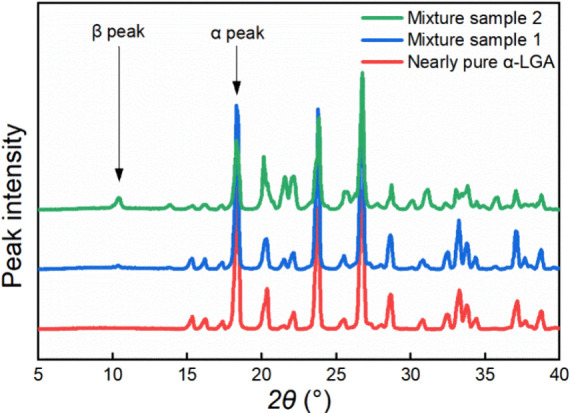
XRD spectra of three representative LGA samples. Nearly pure α-LGA sample is from the semibatch experiment under 3.3 mL/min, mixture sample 1 is from the semibatch experiment under a slow dosing rate of 0.33 mL/min, and mixture sample 2 is from the semibatch experiment under 3.3 mL/min with ultrasound.

Moreover, the desupersaturation profile of ultrasound-assisted semibatch crystallization followed the pattern that a high supersaturation level was maintained for a longer time in the case of individual crystal habit, seen from the supersaturation plateau shown in Figure 19 (d). It is suggested that the presence of ultrasound could reduce the critical supersaturation level required for favoring the rapid growth of the nuclei. Subsequently, growth-dominated crystallization proceeded with one population at a relatively lower supersaturation level, leading to more uniform, less-agglomerated crystals.

Additionally, the application of ultrasound in the batch process was investigated by introducing ultrasound after batch addition of a portion of the acid and combined with a semibatch addition. Based on the results in Section 3.3, the induction time under the condition of 20% batch acid was much longer than under the condition of 40% batch acid, while at the condition of 10% acid there was no crystallization occurring in 24 h. Therefore, ultrasound was applied after batch addition of 20% acid and 10% acid, respectively. Three trials were conducted: a) 20% acid +3.3 mL/min, b) 20% acid +1.1 mL/min, and c) 10% acid +1.1 mL/min. The average induction time under the condition with 20% batch acid decreased by 75.9% (from 30.3 to 7.3 min). Ultrasound was capable of inducing nucleation under the condition of 10% batch acid, as seen in the BlazeMicro images and confirmed by microscopy analysis. However, the slurry under 10% acid was too thin to give a significant increase in Blaze turbidity. The significant increase in turbidity corresponding to the burst nucleation occurred during the subsequent semibatch dosing period. In the end, well-structured intergrown crystals were obtained in all three trials with a similar particle size and PSD, as shown in Figure 20, suggesting the reproducibility of the hybrid strategy in controlling crystal properties.

#### 3.4.2 Deagglomeration effects

In this section, deagglomeration effects were investigated by applying ultrasound to the slurry prepared by resuspending the experimentally collected intergrown agglomerates in the mother liquor. Figure 21 (a) – (b) shows the BlazeMicro images of the slurry from which the collected crystals are produced and the slurry resuspended for the deagglomeration tests using the collected dry products, respectively. The concentration of mother liquor was monitored by the ATR-FTIR probe, and there was no change in the concentration, suggesting that no dissolution or crystallization occurred during the deagglomeration experiment. XRD examination showed that α-LGA crystals were recycled after the deagglomeration tests, meaning there were no effects of this certain condition of ultrasound on the polymorph. The deagglomeration process was monitored by the Blaze probe, and the particle counts based on chord length and chord length-based distribution (CLD) was analyzed.

One example is demonstrated in Figure 21 (c) – (d), together with the offline PSD comparison in Figure 21 (e). It was seen from the BlazeMicro inline images, the CLD trends, and the particle count trends that ultrasound significantly deagglomerated the intergrown crystals. The small crystals tended to agglomerate again after ultrasound was switched off, as seen from both the CLD peak changes and the particle count trends, where small particles decreased and large particles increased. This can support the idea that the crystals of smaller sizes have a higher tendency to agglomerate, the same observation as in the crystallization experiments that led to intergrown crystal habits. After three cycles of ultrasound on–off, the CLD trends and particle counts reached a result close to that of the second cycle and the peak of large size disappeared, which indicated that it reached the limitation of the deagglomeration effects for that certain ultrasound condition. It is expected that higher power of ultrasound would further reduce the crystal sizes, even though it was not the objective here, about which the reader may refer to the literature.^52^ One can conclude that ultrasound could deagglomerate the intergrown α-LGA crystals and reduce the particle sizes without altering the polymorph or dissolving the smaller crystals.

## 4 General discussion

### 4.1 The overall impact of dosing strategies

In this study, reactive crystallization of l-glutamic acid was carried out by dosing sulfuric acid into the initial MSG solution. The experiments were conducted under a constant temperature and using the same concentration and total volume. Various dosing strategies, including batch addition, semibatch addition with constant rates and two-step rates, and hybrid batch/semibatch addition, were investigated.

The solid yields, polymorphism, crystal habits, and crystal sizes were evaluated. The crystallization yields are almost the same, attributed to the same reaction equilibrium state. Regarding the polymorphic form of LGA, most experiments favor α-LGA, indicating its kinetic advantage. However, there were exceptions. For instance, under low dosing rates of 0.3 mL/min, a mixture of both crystal forms was obtained due to the long crystallization time, increasing the chance for solution-mediated conversion to the thermodynamically stable form of β-LGA. Figure 22 shows the XRD spectra of three experimentally produced LGA samples, which represent nearly pure α-form LGA and mixtures of both forms mentioned in this study. The semibatch experiments under fast-consumption-slow dosing conditions also resulted in the formation of β in the second slow-dosing period. Therefore, keeping the process for a longer period of time in the presence of formed α-LGA crystals could increase the chance of producing β-LGA due to solution-mediated transformation to the stable form.

From the perspective of crystal habits of α-LGA, two distinct crystal habits were found – either individual crystals or intergrown agglomerates. Rather than judging the relative advantages of the crystal habits, this study focuses on exploring their relationship with supersaturation. There seems to be a critical supersaturation level S_crt_ under the semibatch condition, which influences the initial crystal habits and controls the final dominant crystal habits. It is found that once the supersaturation level at the nucleation onset is above S_crt_, it favors the rapid growth of nuclei and leads to large individual crystals. The supersaturation level at nucleation onset which is below S_crt_ cannot provide sufficient driving force for rapid growth, and the small crystals collide with each other, resulting in the intergrown agglomerates. Similar intergrown phenomenon was reported by Jirát et al.^53^ Neither decreasing nor increasing the dosing rates after the initial supersaturation consumed could change the dominant crystal habit. However, increasing the dosing rates could increase the supersaturation level and favor secondary nucleation, leading to an increase in the number of small individual crystals. Regarding the particle size, the cases with intergrown agglomerates appear to have narrower PSD spans compared with the cases with individual crystals, despite the fact that there were no significant differences in the average volume-based sizes.

Overall, the semibatch process is more controllable by controlling the supersaturation generation profile compared to the batch process. However, the induction points of semibatch processes at relatively high dosing rates can be stochastic, leading to stochastic crystal habits of nearly pure metastable α-LGA. The semibatch processes at too low dosing rates may yield a mixture of metastable and stable forms. Moreover, the hybrid batch/semibatch crystallization process combines predefined supersaturation for nucleation onset with continuous dosing of the remaining acid to complete the reaction. First batch addition of acid enables nucleation, while subsequent continuous dosing promotes the controlled growth of well-structured α-LGA crystals. This strategy reduces the reaction intensity of batch dosing all reactants and avoids the stochasticity of the crystal habits typical of semibatch processes, which suggests its advantage in controlling both the nucleation onset and the final crystal properties. As shown in Figure 23, both conventional and ultrasound-assisted hybrid processes produced crystals with narrower PSDs compared to the batch process. The semibatch process was excluded for comparison from Figure 23 due to its strong variability in PSD caused by stochastic crystal habits.

**Figure 23:**
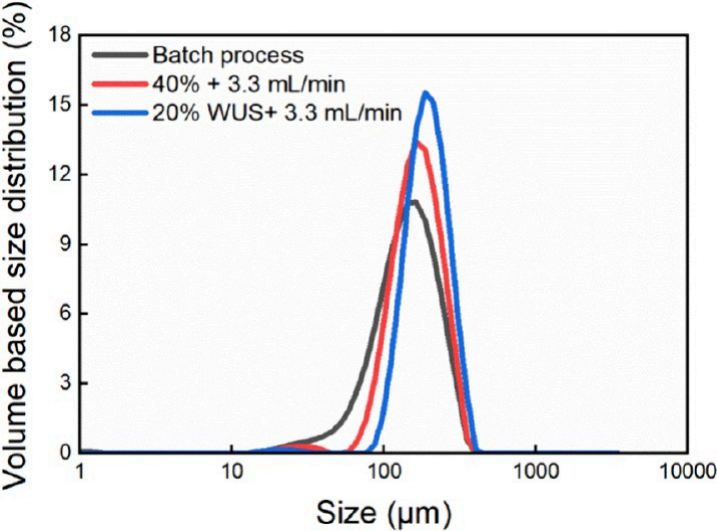
PSD comparison of samples produced by the batch process and hybrid processes. The PSD results from the semibatch process can be varied depending on the stochastic crystal habits, therefore not included here.

### 4.2 The potential role of ultrasound in reactive crystallization

The presence of ultrasound can affect the competition among nucleation, growth, and agglomeration. In this work, ultrasound was found to make the induction time shorter and more reproducible during both semibatch and batch reactive crystallization of LGA. Nevertheless, a certain condition of ultrasound applied in this work could not improve the repeatability of the crystal habits. In some sonicated semibatch experiments, ultrasound led to a polymorphic mixture dominated by metastable α-LGA with trace amounts of stable β-LGA. This is because the ultrasound-enhanced nucleation, which is not form-specific, allows occasional β-LGA nucleation but does not overcome the kinetic advantage of the metastable α-LGA. The hybrid process, during which ultrasound is applied in the batch stage to reduce the induction time and coupled with a semibatch stage, led to an efficient and controlled crystallization process and well-structured intergrown crystals. Additionally, applying ultrasound in the saturated slurry of intergrown agglomerates reduced the average crystal sizes while maintaining the polymorphism. Therefore, ultrasound can potentially reduce the induction time, enhance the crystallization kinetics, affect the competition of different crystal forms, and reduce the final particle size. It seems to be preferred to apply ultrasound in the hybrid process from the perspective of controlling the reactive crystallization process and the final products.

## 5 Conclusions

Using l-glutamic acid as a model compound, this study explored the effects of different supersaturation generation profiles on the reactive crystallization process. The integration of process analytical technologies (PATs) such as in situ microscopy and ATR-FTIR allowed real-time tracking of the supersaturation level and the nucleation and growth behavior. The experimental findings demonstrate that the supersaturation level and its generation profile significantly affect the competition between nucleation and growth, leading to different crystal habits, crystal size distributions, and polymorph composition. Furthermore, the introduction of ultrasound contributed to a shortened and highly reproducible induction time in both batch and semibatch processes. Ultrasound showed promising potential in enhancing crystallization kinetics of both the α- and β-forms, thus increasing the likelihood of producing a polymorphic mixture. These results highlight the value of PAT-enabled crystallization monitoring for studying the crystallization process and the importance of understanding the role of supersaturation profiles in designing robust reactive crystallization processes. Future work will focus on further investigating the interplay between supersaturation profiles and polymorphic forms and optimizing ultrasound parameters for polymorph control.

## Supplementary Material



## Notes

S1: Turbidity data processing for induction time detection, including raw measurement of turbidity, normalized data, smoothed data, and the first derivation data; S2: Detailed conditions of ATR-FTIR experiments; S3: The behavior of probes when ultrasound is on (PDF)
