# Supplementary material for: Study of Batch and Semibatch Reactive Crystallization of l‑Glutamic Acid with aid of PATs, focusing on Polymorphism and Crystal Habit
Source: Cryst Growth Des. 2025 Oct 15;25(21):9107–24. doi: 10.1021/acs.cgd.5c01005 (PMC12593399; doi:10.1021/acs.cgd.5c01005)
Supplement: Supplementary file 1 [file cg5c01005_si_001.pdf]

Supporting Information

Study of Batch and Semi-batch Reactive Crystallization of L-Glutamic Acid with aid of PATs, focusing on Polymorphism and Crystal Habit

Biyu Zhang[a], Christos Xiouras[b], Merve Öner[b], Georgios D. Stefanidis[c], Tom Van Gerven[a,]*

a,* Department of Chemical Engineering, Process Engineering for Sustainable Systems, KU Leuven, Celestijnenlaan 200F, 3001 Leuven, Belgium

b Janssen Research and Development, Janssen Pharmaceutical Companies of Johnson & Johnson, Turnhoutseweg 30, 2340 Beerse, Belgium

c School of Chemical Engineering, Department of Process Analysis and Plant Design, National Technical University of Athens, Iroon Polytecneiou 9, Zografou 15780, Athens, *Greece*

**Corresponding author: tom.vangerven@kuleuven.be*

S1. Turbidity data processing for induction time detection

This section provides a representative example of the turbidity data processing used to identify the nucleation point. The raw Blaze TU data was normalized and smoothed in MATLAB, followed by calculation of the first derivative. The nucleation point was determined as the first data point where the rate of change exceeded a predefined threshold. Figure S1 presents the raw, normalized, smoothed, and first derivative curves for this example.

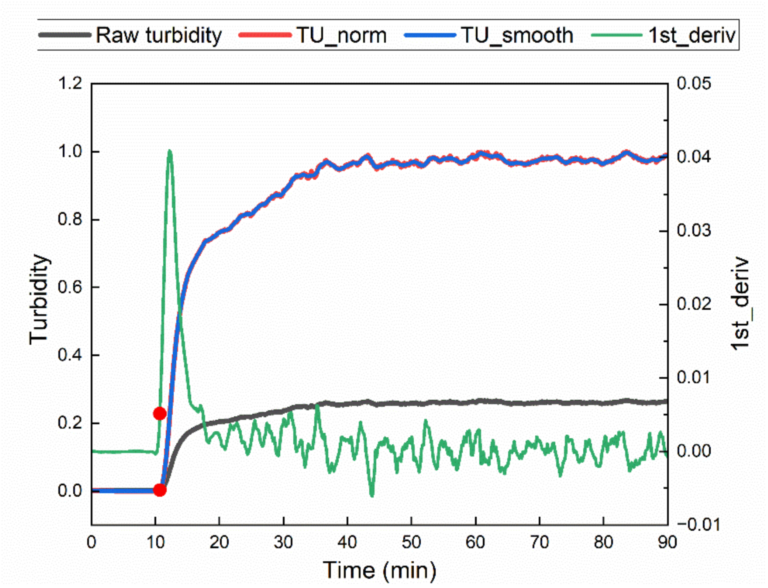

Figure S1. Example of turbidity data processing for induction time detection. Experiment condition used in this figure: initial MSG 200 mL of 1.0M, dosing sulfuric acid 200 mL of 0.5M at 6.7 mL/min.

S2. ATR-FTIR calibration experiments

Both equilibrium experiments and dynamic crystallization experiments were conducted for ATR-FTIR calibration. The equilibrium experiment was carried out stepwise adding sulfuric acid to the preheated monosodium glutamate solution. A total amount of 200 mL was added in ten steps. After each addition, an offline sample was taken, and after holding overnight for equilibrium, the other sample was taken. The corresponding IR spectra was collected, and the concentration of offline samples was analyzed in UPLC (ultra-performance liquid chromatography). Similarly, the dynamic crystallization experiments under different dosing rates were conducted. The solution samples at different time points were used as the model input, together with the corresponding IR spectra. The experimental conditions are shown in Table S1.

Table S1. Detailed conditions of ATR-FTIR calibration experiments

IR calibration experiments	Dosing rate (mL/min)	Dosing duration (min)	Holding time (h)	Number of samples taken
dynamic experiments	6.7	30	1	4
	6.7	30	1	3
	6.7	30	24	12
	3.3	60	24	8
	3.3	60	4	6
	3.3	60	1	2
	1.7	120	24	9
	1.1	180	24	13
equilibrium experiment	stepwise dosing 20 mL at once via a funnel		holding overnight after each dosing	18

Using the above data, the IR calibration model was built using the software iC Quant. The settings of iC Quant and the model information are shown in Table S2.

Table S2. iC Quant settings and IR model information

Model type	multivariate
Algorithm applied	partial least squares regression
Predictor	IR spectra, specified region 1500 cm^{-1} – 1000 cm^{-1}
Response	Total concentration [Glu]
Data processing	Mean center
Maximum factors	20

Optimal factors	7
R^2 cumulative	0.993
Root mean square error of Calibration (RMSEC)	0.0283
Root mean square error of cross validation (RMSECV)	0.034

S3. Effects of ultrasound on the initial solution and PATs

The condition – 40W, 24 kHz, 0.5-cycled pulse ultrasound (0.5s sonication - 0.5s pause) was applied in all ultrasound experiments. Before applying ultrasound in the crystallization experiments, the effects on the initial solution and PATs were investigated, including the temperature, pH, IR spectra, and Blaze turbidity. As shown in Figure S2, during the 10-minute ultrasound, the temperature was maintained around 25 ±1 °C. No influence on pH and IR spectra. The turbidity had a slight increase from 0.0016 to 0.0017 and remained at 0.0017 after 2 minutes, negligible compared to the final turbidity of crystallization experiments of up to 0.3. Therefore, the effects of ultrasound on the process measurements were negligible.

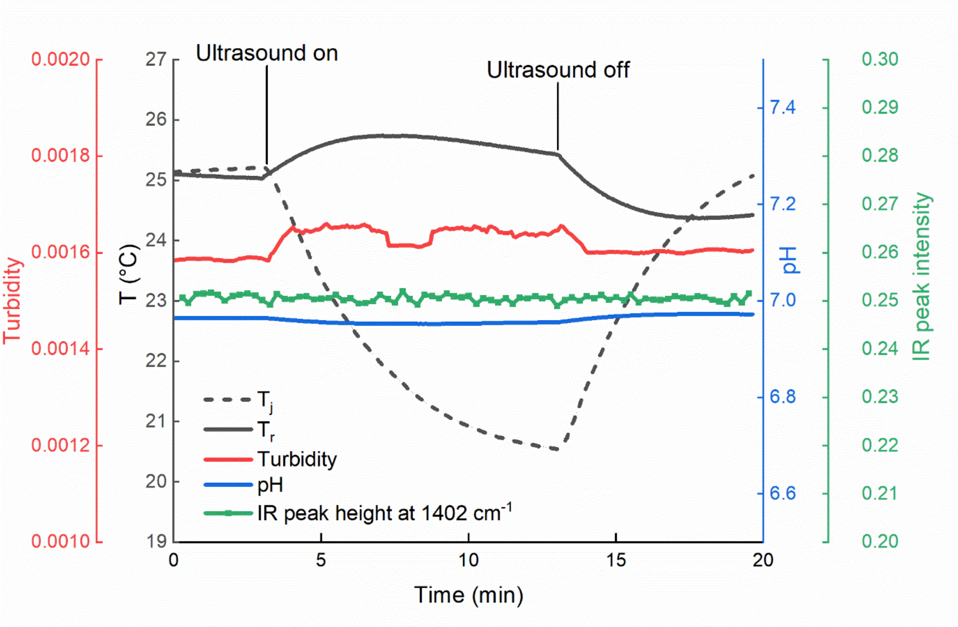

Figure S2. Effects of 10-minute ultrasound on initial solution before dosing acid. T_j and T_r are the temperature of jacket and reactor, respectively. IR peak at 1402 cm^{-1} is the characteristic peak of carboxylate.
